# *Bifidobacterium bifidum* Extracellular Sialidase Enhances Adhesion to the Mucosal Surface and Supports Carbohydrate Assimilation

**DOI:** 10.1128/mBio.00928-17

**Published:** 2017-10-03

**Authors:** Keita Nishiyama, Yuji Yamamoto, Makoto Sugiyama, Takashi Takaki, Tadasu Urashima, Satoru Fukiya, Atsushi Yokota, Nobuhiko Okada, Takao Mukai

**Affiliations:** a Department of Microbiology, School of Pharmacy, Kitasato University, Tokyo, Japan; b Department of Animal Science, School of Veterinary Medicine, Kitasato University, Towada, Aomori, Japan; c Faculty of Veterinary Medicine, School of Veterinary Medicine, Kitasato University, Towada, Aomori, Japan; d Section of Electron Microscopy, Showa University, Tokyo, Japan; e Graduate School of Animal and Food Hygiene, Obihiro University of Agriculture and Veterinary Medicine, Obihiro, Hokkaido, Japan; f Research Faculty of Agriculture, Hokkaido University, Sapporo, Hokkaido, Japan; University of Queensland

**Keywords:** adhesion molecules, bacterial adhesion, bifidobacteria, carbohydrate metabolism, sialidase

## Abstract

*Bifidobacterium* is a natural inhabitant of the human gastrointestinal (GI) tract. We studied the role of the extracellular sialidase (SiaBb2, 835 amino acids [aa]) from *Bifidobacterium bifidum* ATCC 15696 in mucosal surface adhesion and carbohydrate catabolism. Human milk oligosaccharides (HMOs) or porcine mucin oligosaccharides as the sole carbon source enhanced *B. bifidum* growth. This was impaired in a *B. bifidum* ATCC 15696 strain harboring a mutation in the *siabb2* gene. Mutant cells in early to late exponential growth phase also showed decreased adhesion to human epithelial cells and porcine mucin relative to the wild-type strain. These results indicate that SiaBb2 removes sialic acid from HMOs and mucin for metabolic purposes and may promote bifidobacterial adhesion to the mucosal surface. To further characterize SiaBb2-mediated bacterial adhesion, we examined the binding of His-tagged recombinant SiaBb2 peptide to colonic mucins and found that His-SiaBb2 as well as a conserved sialidase domain peptide (aa 187 to 553, His-Sia) bound to porcine mucin and murine colonic sections. A glycoarray assay revealed that His-Sia bound to the α2,6-linked but not to the α2,3-linked sialic acid on sialyloligosaccharide and blood type A antigen [GalNAcα1-3(Fucα1-2)Galβ] at the nonreducing termini of sugar chains. These results suggest that the sialidase domain of SiaBb2 is responsible for this interaction and that the protein recognizes two distinct carbohydrate structures. Thus, SiaBb2 may be involved in *Bifidobacterium*-mucosal surface interactions as well as in the assimilation of a variety of sialylated carbohydrates.

## IMPORTANCE

Adhesion to the host mucosal surface and carbohydrate assimilation are important for bifidobacterium colonization and survival in the host gastrointestinal tract. In this study, we investigated the mechanistic basis for *B. bifidum* extracellular sialidase (SiaBb2)-mediated adhesion. SiaBb2 cleaved sialyl-human milk oligosaccharides and mucin glycans to produce oligosaccharides that supported *B. bifidum* growth. Moreover, SiaBb2 enhanced *B. bifidum* adhesion to mucosal surfaces via specific interactions with the α2,6 linkage of sialyloligosaccharide and blood type A antigen on mucin carbohydrates. These findings provide insight into the bifunctional role of SiaBb2 and the adhesion properties of *B. bifidum* strains.

## INTRODUCTION

The human intestinal microbiota consists of 10^13^ to 10^14^ microorganisms that form a highly complex microbial ecosystem (1). Bifidobacteria are high-G+C, Gram-positive bacteria of the family *Bifidobacteriaceae*, class *Actinobacteria*. Bifidobacterial colonization of the human gastrointestinal (GI) tract occurs shortly after delivery; however, abundance declines with age such that this genus represents <10% of the human adult microbiota (2–4). Some species, such as *Bifidobacterium bifidum*, *Bifidobacterium breve*, *Bifidobacterium longum* subsp. *infantis*, and *Bifidobacterium longum* subsp. *longum*, are specific to the human gut and are some of the predominant members of the gut microbiota in breastfed infants (3, 4). *B. bifidum* and *B. longum* subsp. *longum* are also present in adult humans and are considered key commensals that promote multiple host functions, including immune system modulation (5) and protection against pathogens (5, 6).

The cell surface of Gram-positive bacteria—which is composed of a peptidoglycan layer, teichoic acids, proteins, and exopolysaccharide—comprises species- and strain-specific molecules. Most of these function as adaptive and probiotic factors since they are in direct contact with the environment. Cell surface proteins can function as adhesion factors (e.g., in biofilm formation), antigens, receptors, enzymes, and transporters (reviewed in references 7 and 8).

The mucus layer of the human gastrointestinal (GI) tract varies in thickness from 30 to 600 µm, becoming thicker from the small intestine to the rectum (9). Mucin secretion and turnover create a fluid environment in the GI tract for the movement of gut contents. The mucus layer also provides a habitat for microbiota and is the first point of contact between intestinal microbiota and the host (10). As such, adhesion to the mucosal surface is a prerequisite for the colonization and persistence of nonmotile organisms (e.g., bifidobacteria) in the GI tract and provides a competitive advantage in this ecosystem. Some *Bifidobacterium* strains express adhesins on their cell surface that mediate attachment to the mucus layer (11–17). Pili or fimbriae are often required for adhesion of and host colonization by *Bifidobacterium*; type IVb or tight adherence (Tad) pili from *B. breve* UCC2003 (12) and sortase-dependent pili from *B. bifidum* PRL2010 (15) serve these functions in the murine gut. The fimbria-associated BL0675 subunit found in several *B. longum* subsp. *longum* strains (16) promotes adhesion to mucin (17).

*Bifidobacterium* species reside in the large intestine, where there is a low abundance of sugars; their survival and growth therefore require a variety of extracellular and cytoplasmic glycosyl hydrolases that hydrolyze indigestible oligosaccharides (reviewed in references 18 to 20). Interestingly, *Bifidobacterium* species in infants have unique metabolic pathways for free human milk oligosaccharides (HMOs) and/or intestinal glycoconjugates (e.g., mucin glycan). *B. breve*, *B. longum* subsp. *infantis*, and *B. longum* subsp. *longum* have ATP-binding cassette transporters for internalization of intact oligosaccharides (16, 18–20), which are subsequently degraded by intracellular glycosyl hydrolases that target linkages specific to HMOs and mucin glycans (20). In contrast, *B. bifidum* produces several extracellular glycosyl hydrolases, such as α-l-fucosidase (21, 22), lacto-*N*-biosidase (23), β-galactosidase (24), and β-*N*-acetylhexosaminidase (24), that cleave HMOs and mucin glycans into mono- or disaccharide units. Additionally, endo-α-*N*-acetylgalactosaminidase and α-*N*-acetylgalactosaminidase release oligosaccharides from mucin *O*-glycans (25, 26).

Sialic acids (*N*-acetylneuraminic acid [Neu5Ac]) are frequently found at the nonreducing termini of sugar chains on various eukaryotic glycoconjugates such as glycoproteins and glycosphingolipids. Mucin oligosaccharides are most abundant in intestinal glycoconjugates and are frequently modified by sialic acid sugar residues via α2,3 or α2,6 linkages (27). Healthy adults have 300 μg of sialic acid per mg of colonic mucin (28). Sialic acid residues are also added to the terminal positions of HMOs to form sialyllactoses and monosialyl and disialyl lacto-*N*-tetraoses; approximately 16% of HMOs are sialylated (29). Owing to their surface-exposed location and negative charge, sialic acid-containing carbohydrates are resistant to several bacterial glycosidases. Removal of sialic acid from HMOs and intestinal glycoconjugates exposes the glycan moiety, which can then be catabolized (30).

Hydrolytic sialidases (commonly referred to as neuraminidases) usually have broad substrate specificity and cleave α2,3-, α2,6-, and α2,8-linked terminal sialic acids. Sialidases have been identified in the genomes of some *Bifidobacterium* strains, including *B. longum* subsp. *infantis* ATCC 15697 (31), *B. breve* UCC2003 (32), and *B. bifidum* PRL 2010 (33). Extracellular exo-α-sialidases (SiaBb1 and SiaBb2) from *B. bifidum* JCM1254 (34) and intracellular exo-α-sialidases (NanH1 and NanH2) from *B. longum* subsp. *infantis* ATCC 15697 (35) have been shown to degrade sialylated carbohydrates (34, 35). Thus, sialidase activity is thought to play an important role in bifidobacterial catabolism of sialylated HMOs and mucin glycans. However, the function of *Bifidobacterium* sialidase during bacterial growth is unknown. Bifidobacterial sialidases belong to glycoside hydrolase family 33 according to the CAZy classification (34). They are known to function as adhesion factors in several pathogenic bacteria and promote host-bacterium interactions and biofilm formation. For example, neuraminidase A (NanA) of *Streptococcus pneumoniae* is associated with colonization (34, 35), blood-brain barrier penetration (36), and invasion into brain microvascular endothelial cells (37), while the *Pseudomonas aeruginosa* homolog facilitates mucosal infection via biofilm formation (38). However, there is no evidence that bifidobacterial sialidase participates in adhesion to the mucosal surface.

We speculated that extracellular sialidase from *B. bifidum* acts as a bifunctional extracellular glycosidase that modulates *Bifidobacterium*-mucosal surface interactions and assimilation of HMOs and intestinal glycoconjugates. To test this hypothesis, we analyzed the activities of SiaBb2 as an adhesion factor and carbohydrate-degrading enzyme. Our results demonstrate that SiaBb2 promotes *B. bifidum* adhesion to mucins and contributes to HMO and intestinal glycoconjugate assimilation.

## RESULTS

### Cell surface-associated *B. bifidum* SiaBb2 is required for degradation of sialyloligosaccharides.

**FIG 1:**
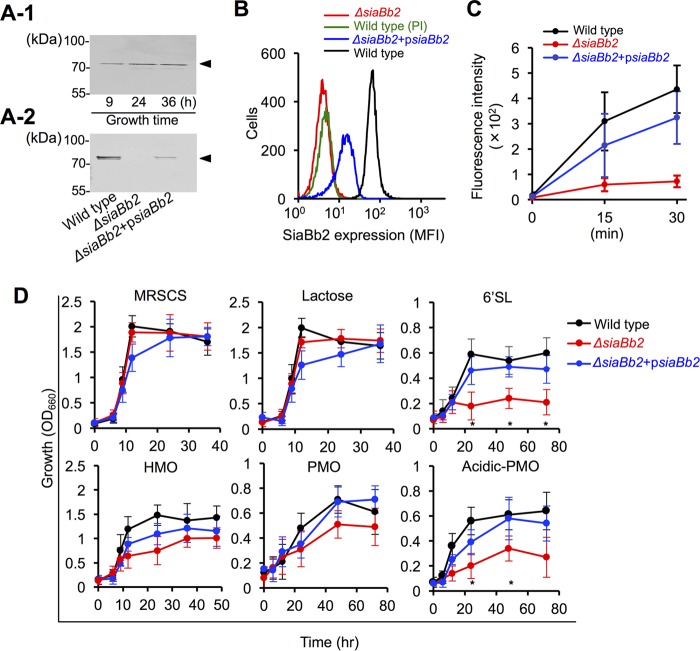
SiaBb2 promotes *B. bifidum* ATCC 15696 degradation of sialyloligosaccharides. (A) Whole-cell lysates (10 μg protein) from the wild-type strain at different culture times (A-1) or from the *siabb2* mutant (*Δsiabb2*) or *siabb2* complementation (*Δsiabb2*+p*siabb2*) strain cultured for 12 h (A-2) were analyzed by Western blotting. Reactivity with anti-SiaBb2 antibody is shown. (B) Surface expression of SiaBb2 in *B. bifidum* ATCC 15696 strains was detected by flow cytometry using an anti-SiaBb2 antibody after 12 h of culture. Preimmune (PI) serum was used for background control. MFI, mean fluorescence intensity. (C) Quantitative analysis of sialidase activity using 2′-(4-methylumbelliferyl)-α-d-*N*-acetylneuraminic acid (4-MU-Neu5Ac). *B. bifidum* (12-h culture) was cultured in the presence of 4-MU-Neu5Ac, and fluorescence was recorded for 30 min. Data represent means ± standard deviations (*n* = 5). (D) Growth profiles of *B. bifidum* in mMRSCS medium supplemented with 1% (wt/vol) lactose, HMO, PMO, 6'SL, or acidic PMO as the sole carbon source. MRSCS medium was used as a positive control. Data represent means ± standard deviations (*n* = 3). *, *P* < 0.05 (one-way analysis of variance followed by Tukey’s honestly significant difference *post hoc* test in pairwise comparisons of wild-type and Δ*siabb2* strains at each time point).

**FIG 2:**
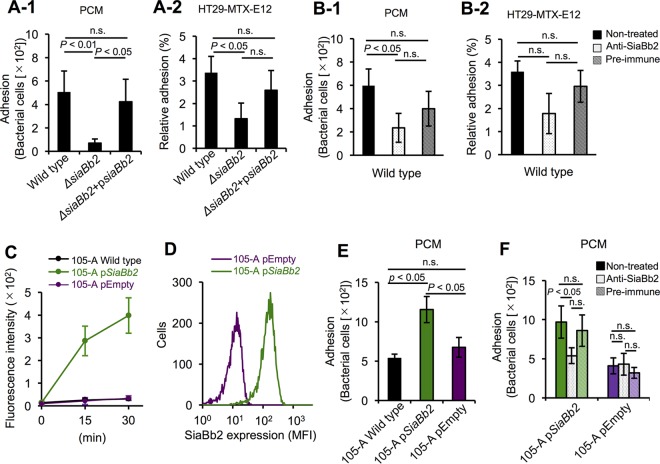
SiaBb2 promotes bifidobacterial adhesion to the mucosal surface. (A) Adhesion of *B. bifidum* ATCC 15696 strains (wild type, *Δsiabb2*, and *Δsiabb2*+p*siabb2*) to PCM (A-1) or HT29-MTX-E12 epithelial cells (A-2). Bacterial strains (12-h culture) were grown in MRSCS broth. (A-1) Adhesion of *B. bifidum* to PCM as assayed using diagnostic glass slides. Bound bacteria were stained with methylene blue, and randomly selected fields were imaged with a microscope. (A-2) Cells were added to monolayers of epithelial cells. Adherent bacteria were plated on MRSCS agar and counted. (B) Effect of anti-SiaBb2 antibody on the adhesion of wild-type *B. bifidum* ATCC 15696 cells to PCM (B-1) or HT29-MTX-E12 epithelial cells (B-2). Bacteria were incubated with anti-SiaBb2 antibody or rabbit preimmune serum (1:100 dilution). (C) Analysis of sialidase activity. *B. longum* subsp. *longum* 105-A strains (105-A wild type, pKKT427-Cm^r^-*siabb2*-transformed strain [105-A p*siabb2*], and empty vector control [105-A pEmpty]) were incubated with 4-MU-Neu5Ac, and fluorescence was recorded for 30 min. Data are presented as means ± standard deviations (*n* = 4). (D) Cell surface expression of SiaBb2 of *B. longum* subsp. *longum* 105-A strains was confirmed by flow cytometry using an anti-SiaBb2 antibody. (E) Adhesion of *B. longum* subsp. *longum* 105-A strains to PCM. (F) Effect of anti-SiaBb2 antibody or rabbit preimmune serum on adhesion of *B. longum* subsp. *longum* 105-A strains to PCM. (A, B, E, and F) Data are presented as means ± standard deviations (*n* = 5). *P* < 0.05 (one-way analysis of variance followed by Tukey’s honestly significant difference *post hoc* test in pairwise comparisons among strains). n.s., nonsignificant.

**FIG 3:**
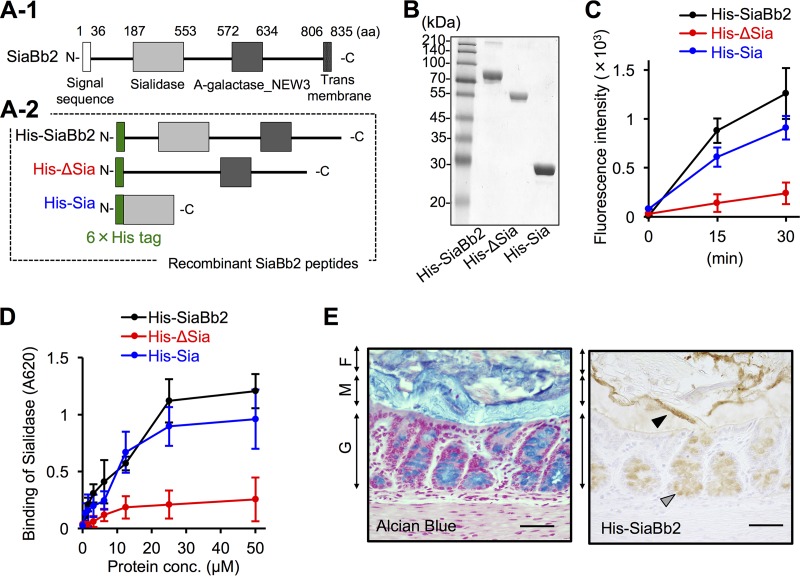
Binding properties of recombinant sialidase peptides. (A-1) Schematic representation of the primary structure of SiaBb2 from *B. bifidum* ATCC 15696. Amino acid numbering starts at the presumed initiation codon. Sialidase and alpha-galactosidase NEW3 (A-galactosidase_NEW3) domains are depicted as light and dark gray boxes, respectively. The white box at the N terminus and the gridded box at the C terminus indicate a signal peptide and membrane anchor, respectively. (A-2) Schematic representation of SiaBb2-truncated peptides expressed as fusions to 6×His tag at the N terminus (green boxes). (B) Purified SiaBb2-truncated peptides (10 μg per lane) from *B. bifidum* ATCC 15696 were separated by sodium dodecyl sulfate-polyacrylamide gel electrophoresis and visualized by staining with Coomassie brilliant blue. The molecular mass standard is shown on the left. (C) Sialidase activity of SiaBb2-truncated proteins. Recombinant proteins (50 μM) were incubated with 4-MU-Neu5Ac, and fluorescence was recorded for 30 min. Data are presented as means ± standard deviations (*n* = 5). (D) Binding of SiaBb2-truncated peptides to PCM, as determined by enzyme-linked immunosorbent assay. PCM-coated wells were incubated with 0.025 to 50 μM recombinant peptide. Binding was detected with an anti-His antibody. Data are presented as means ± standard deviations (*n* = 3). (E) His-SiaBb2 binding to murine colonic tissue sections. Total glycoconjugates were detected by alcian blue staining. Biotinylated His-SiaBb2 specifically reacted with the mucosal surface at the border between the mucus layer and fecal material (black arrowhead) and with goblet cells (gray arrowhead). F, fecal material; M, mucus layer; G, goblet cells. Bars, 200 μm.

*siabb2* from *B. bifidum* ATCC 15696 has a 2,508-bp open reading frame and encodes an 835-amino-acid (aa) polypeptide containing an N-terminal Sec signal sequence and C-terminal transmembrane domain (see Fig. 3A-1). SiaBb2 expression was abundantly detected in *B. bifidum* ATCC 15696 whole-cell extracts throughout the culture period by Western blotting (Fig. 1A-1). Cell surface expression of SiaBb2 was analyzed by flow cytometry in cells cultured for 12 h. Strong fluorescence was detected in *B. bifidum* cells but not in preimmune serum (Fig. 1B), confirming the specificity of the signal. The cell suspension also exhibited sialidase activity (Fig. 1C). These results indicate that SiaBb2 is a cell surface-localized extracellular enzyme. To investigate SiaBb2 function(s) in *B. bifidum*, we disrupted the *siabb2* gene by homologous recombination (see Fig. S1A in the supplemental material). Targeted disruption of *siabb2* in the mutant strain was confirmed by Western blotting (Fig. 1A-2), Southern hybridization analysis (Fig. S1B), and PCR (Fig. S1C). There were no differences in the major bands between wild-type and *Δsiabb2* whole-cell lysates (Fig. S1D). However, the *Δsiabb2* strain showed markedly decreased cell surface fluorescence (Fig. 1B) and enzymatic activity (Fig. 1C), which were restored to wild-type levels by introducing a p*siabb2* expression plasmid (*Δsiabb2*+p*siabb2*, complementation strain) (Fig. 1A-2, B, and C).

10.1128/mBio.00928-17.2 FIG S1  Targeted disruption of the *B. bifidum* ATCC 15696 *siabb2* gene by homologous recombination. (A) Schematic representation of the genomic structure of the *B. bifidum* ATCC 15696 *siabb2* gene (GenBank accession no. LC228603), the targeting vector pBS423-*ΔrepA*, and the resultant mutant generated by a single-crossover event. (B) Southern hybridization analysis of genomic DNA from wild-type and *Δsiabb2* strains. Samples of EcoRI- and EcoRV-digested chromosomal DNA were resolved by agarose gel electrophoresis. Probe 1, depicted as a blue box in panel A, was used to detect the ~5.7-kb (wild-type) EcoRI/EcoRV and ~10-kb (*Δsiabb2*) EcoRI/EcoRV genomic fragments. Probe 2, depicted as a purple box in panel A, was used to detect the *sp.R* gene containing EcoRI/EcoRV and ~10-kb (*Δsiabb2*) EcoRI/EcoRV genomic fragments. Sizes of hybridized fragments are indicated to the left of the panel. (C) PCR analysis of genomic DNA from wild-type and *Δsiabb2* strains. The PCR product for the wild-type *siabb2* gene was 2.5 kb. For the mutant generated by the single-crossover event, the PCR products were ~6.9 kb (*siabb2* gene) and 0.6 kb (*sp.R* gene), respectively. PCR primer positions are shown in panel A. (D) Coomassie brilliant blue-stained sodium dodecyl sulfate-polyacrylamide gel of wild-type and *Δsiabb2* whole-cell lysates. Equal amounts of protein were loaded in each lane. Download FIG S1, TIF file, 1.7 MB. Copyright © 2017 Nishiyama et al. 2017 Nishiyama et al. This content is distributed under the terms of the Creative Commons Attribution 4.0 International license.

We next examined the growth of *B. bifidum* ATCC 15696 in mMRSCS broth (see Materials and Methods) supplemented with HMO or porcine mucin oligosaccharide (PMO) as a carbon source by measuring the optical density of the cultures (Fig. 1D). Wild-type and *Δsiabb2* mutant strains grew rapidly in MRSCS broth, with cell density reaching a maximum (optical density at 660 nm [OD_660_] of ~2.0) within 15 h of incubation; however, the cell density of Δ*siabb2*+p*siabb2* cultures was slightly lower (Fig. 1D, MRSCS). The growth patterns were similar when lactose was used as the sole carbon source (Fig. 1D, lactose). HMO or PMO as the sole carbon source supported the growth of the wild-type strain, which entered stationary phase after 24 h (HMO) or 48 h (PMO). The *Δsiabb2* strain showed a growth pattern similar to that of the wild-type strain in the early growth phase (Fig. 1D, HMO and PMO) but grew more slowly after the mid-exponential phase (Fig. 1D, HMO and PMO), although the difference with respect to wild-type cultures was not statistically significant. To investigate whether SiaBb2 is required for sialyl-HMO and -PMO metabolism in *B. bifidum* cells, 6′-sialyllactose (6'SL) or acidic PMO was added as a sole carbon source. As expected, the growth of the *Δsiabb2* strain decreased after 24 h of culture (Fig. 1D, 6'SL and Acidic-PMO). When wild-type and *Δsiabb2* strains were cocultured with HMO as the sole carbon source, there was no difference in their respective cell counts compared to single cultures after 24 h (Fig. S2A), even though the mutant strain could not use sialic acid as a carbon source (Fig. S2B). These results suggest that the wild-type strain supported *Δsiabb2* cell growth via production of desialylated oligosaccharides. A previous study suggested that since extracellular SiaBb2 can remove both α2,3- and α2,6-linked sialic acids, it plays a critical role in bifidobacterial catabolism of sialylated HMOs and mucin glycans (34). Our results indicate that *siabb2* is constitutively expressed in *B. bifidum* ATCC 15696 and promotes the digestion of sialyl-HMO and -PMO to yield utilizable oligosaccharides.

10.1128/mBio.00928-17.3 FIG S2  Growth effect of *B. bifidum* ATCC 15696 on HMO or Neu5Ac as a carbon source. (A) Growth profiles of wild-type and *Δsiabb2 B. bifidum* ATCC 15696 strains cultured in mMRSCS containing 0.5% (wt/vol) HMO. (Right panel) Single-culture model. (Left panel) Coculture model. (B) Growth profiles of wild-type *B. bifidum* ATCC 15696 in mMRSCS containing 0.5% (wt/vol) Neu5Ac. Data are presented as means ± standard deviations (*n* = 3). Significant differences were determined by analysis of variance with the Tukey test. *, *P* < 0.05 versus wild-type and *Δsiabb2* strains. ns, nonsignificant. Download FIG S2, TIF file, 0.6 MB. Copyright © 2017 Nishiyama et al. 2017 Nishiyama et al. This content is distributed under the terms of the Creative Commons Attribution 4.0 International license.

### SiaBb2 promotes *B. bifidum* adhesion to the mucosal surface.

Mucin glycoprotein is a major receptor supporting bacterial colonization of the mucosal surface (2–4). To determine the contribution of SiaBb2 to bacterial adhesion to the mucosa, we used the mucus-secreting HT29-MTX-E12 intestinal cell line, which secretes both MUC5Ac and MUC2 (39). The *Δsiabb2* mutant showed reduced adhesion to immobilized porcine colonic mucin (PCM) (Fig. 2A-1) and HT29-MTX-E12 cells in the late exponential growth phase (12-h culture in MRSCS broth) (Fig. 2A-2) compared to the wild-type strain; adhesion was restored to wild-type levels in the complementation strain (Fig. 2A). In addition, adhesion of wild-type cells to PCM was reduced (Fig. 2B-1), whereas no reduction was observed in the adhesion to HT29-MTX-E12 cells (Fig. 2B-2) following treatment with an anti-SiaBb2 antibody. The rabbit preimmune serum had no effect (Fig. 2B). In addition, anti-SiaBb2 antibody treatment inhibited adhesion of the SiaBb2-expressing *B. bifidum* JCM1254 strain (Fig. S3A) to HT29-MTX-E12 (Fig. S3B-1) and PCM (Fig. S3B-2). These results indicate that SiaBb2 mediates adhesion in *B. bifidum*. However, it is possible that steric hindrance by SiaBb2 antibody nonspecifically bound to other adhesion factors reduced *B. bifidum* adhesion. To exclude this possibility, we examined SiaBb2 expression in *B. longum* subsp. *longum* strain 105-A at the mid-exponential growth phase (12-h culture in MRSCS broth). p*siabb2* expression conferred sialidase activity compared to wild-type and empty-vector control strains (Fig. 2C). The presence of SiaBb2 on the surface of cells harboring p*siabb2* was confirmed by flow cytometry (Fig. 2D). *B. longum* subsp. *longum* 105-A p*siabb2* cells showed increased adhesion to PCM compared to wild-type and empty-vector control strains (Fig. 2E); however, this was reduced in *B. longum* subsp. *longum* 105-A p*siabb2* but not control cells following treatment with the anti-SiaBb2 antibody (Fig. 2F). Similar adhesion patterns were observed in early-exponential-growth-phase cells (9-h culture) (Fig. S3C-1 and C-2). Interestingly, in stationary-growth-phase cells (25-h culture), the degree of adhesion to PCM was unaffected by *siabb2* mutation (Fig. S3D-1) and anti-SiaBb2 antibody treatment (Fig. S3D-2). SiaBb2-associated fluorescence signal was detected in early (9-h) and late (12-h) exponential cells but not in stationary-phase cells (25 h) (Fig. S4A). In contrast, whole-cell sialidase activities were similar across growth phases (Fig. S4A).

10.1128/mBio.00928-17.4 FIG S3  Adhesion properties of *B. bifidum* strains. (A) SiaBb2 was detected in whole-cell lysates (10 μg protein) of *B. bifidum* strains by Western blotting. (B) Effect of anti-SiaBb2 antibody on *B. bifidum* JCM1254 adhesion to HT29-MTX-E12 epithelial cells (B-1) or PCM (B-2). Bacteria were incubated with anti-SiaBb2 antibody or rabbit preimmune serum (1:100 dilution). (C and D) Adhesion of *B. bifidum* ATCC 15696 strains to PCM in the early exponential growth phase (9-h culture) (C) and stationary growth phase (25-h culture) (D). (C-1 and D-1) Adhesion of *B. bifidum* strains (wild type, *Δsiabb2*, and *Δsiabb2*+p*siabb2*) to PCM, as assayed using diagnostic glass slides. (C-2 and D-2) Effect of anti-SiaBb2 antibody on wild-type *B. bifidum* ATCC 15696 adhesion to PCM. Data represent means ± standard deviations (*n* = 3). Download FIG S3, TIF file, 1.1 MB. Copyright © 2017 Nishiyama et al. 2017 Nishiyama et al. This content is distributed under the terms of the Creative Commons Attribution 4.0 International license.

10.1128/mBio.00928-17.5 FIG S4  Cell surface expression and enzymatic activity of SiaBb2 from *B. bifidum* ATCC 15696 during each growth phase. Surface expression was confirmed using an anti-SiaBb2 antibody by flow cytometry (A) and immunoelectron microscopy (B). (A) Upper: quantitative analysis of sialidase activity using 4-MU-Neu5Ac. *B. bifidum* ATCC 15696 cells (10 mg) were incubated with 4-MU-Neu5Ac, and fluorescence was recorded for 30 min. Data are presented as fluorescence intensity ± standard deviation (*n* = 5). Differences were evaluated by one-way analysis of variance followed by the Tukey honestly significant difference *post hoc* test in pairwise comparisons among strains. ns, nonsignificant. (A) (Lower panels) Flow cytometry profiles showing SiaBb2 expression (blue line); preimmune serum was used for background control (black line). (B) Arrowheads indicate gold particle labeling of SiaBb2 protein. Bars, 500 nm. − and +, before and after mutanolysin treatment, respectively. Download FIG S4, TIF file, 2.2 MB. Copyright © 2017 Nishiyama et al. 2017 Nishiyama et al. This content is distributed under the terms of the Creative Commons Attribution 4.0 International license.

The localization of SiaBb2 on the cell surface was examined by transmission electron microscopy. Gold particles labeling SiaBb2 protein were detected in early exponential but not stationary-phase cells (Fig. S4B), suggesting that SiaBb2 was buried in the peptidoglycan layer in the latter phase and was therefore inaccessible to anti-SiaBb2 antibody. To assess this possibility, we performed immunolabeling following mutanolysin digestion of the peptidoglycan layer on stationary-phase cells. SiaBb2-associated fluorescence signal (Fig. S4A) and gold particles were observed on the cell surface (Fig. S4B) but not in preimmune serum (data not shown). These results indicate that SiaBb2 is important for the adhesion of *B. bifidum* to mucin during the active growth phase.

It was previously reported that in *B. longum* subsp. *infantis* ATCC 15697—which uses HMO and harbors *siabb1* and *siabb2* homologs (*nanH1* and *nanH2*, respectively) in its genome (31, 35)—*nanH2* is upregulated in the presence of HMO (35). To determine whether *siabb2* expression is affected by HMO or PMO, we evaluated the level of the gene in *B. bifidum* ATCC 15696. *siabb2* expression was unaltered when HMO or PMO was the sole carbon source (Fig. S5A). In contrast, adhesion of the wild-type strain to PCM was decreased when HMO was used as the sole carbon source, while *siabb2* mutation decreased *B. bifidum* adhesion to PCM in all media (Fig. S5B). The results indicate that the degree of adhesion of *B. bifidum* ATCC 15696 is altered by the carbon source; however, the functionality of SiaBb2 as an adhesion factor may be unaffected.

10.1128/mBio.00928-17.6 FIG S5  *B. bifidum* ATCC 15696 adhesion properties and *siabb2* gene expression during carbohydrate fermentation. Bacterial cells were cultured until late exponential phase with HMO or PMO as the sole carbon source (24 and 36 h, respectively) or in MRSCS (12 h). (A) *siabb2* expression was determined relative to the level in cells grown with HMO or PMO as the sole carbon source compared to those grown in MRSCS. Data represent means ± standard deviations (*n* = 5). Differences were evaluated by one-way analysis of variance followed by the Tukey honestly significant difference (HSD) *post hoc* test in all pairwise comparisons. (B) Adhesion of wild-type strain to PCM, as determined using diagnostic glass slides. A bacterial suspension (OD_600_ of 0.5) was added to wells containing immobilized PCM. Bound bacteria were stained with methylene blue, and randomly selected fields were imaged with a microscope. Data represent means ± standard deviations (*n* = 3). Differences were evaluated by one-way analysis of variance followed by the Tukey HSD *post hoc* test in all pairwise comparisons of strains grown with different carbon sources. Download FIG S5, TIF file, 0.6 MB. Copyright © 2017 Nishiyama et al. 2017 Nishiyama et al. This content is distributed under the terms of the Creative Commons Attribution 4.0 International license.

### The sialidase domain of SiaBb2 is essential for mucin binding.

*B. bifidum* also encodes a 1,795-aa extracellular SiaBb1 protein with a conserved N-terminal Sec signal sequence and C-terminal transmembrane region (Fig. S6A-1) (33). To further characterize SiaBb2-mediated *B. bifidum* ATCC 15696 adhesion, we examined the binding of recombinant N-terminal hexahistidine (His)-tagged SiaBb2 peptides covering different regions of SiaBb2 (i.e., His-SiaBb2, His-ΔSia, and His-Sia [Fig. 3A-2]). The sialidase-truncated peptides produced single bands in the sodium dodecyl sulfate-polyacrylamide gel electrophoresis analysis (Fig. 3B and S6A-2). A fluorescence-based enzyme assay revealed that all sialidase peptides with the exception of His-ΔSia showed time-dependent enzymatic activity (Fig. 3C and S6A-3).

10.1128/mBio.00928-17.7 FIG S6  Purification and biochemical properties of recombinant sialidase proteins (A and B) and their binding to PCM (C). (A-1) Schematic illustration of the primary structure of SiaBb1 from *B. bifidum* ATCC 15696. Each domain is depicted as a box. The white box at the N terminus and gridded box at the C terminus indicate a signal peptide (SS) and membrane anchor (TM), respectively. (A-2) Purified SiaBb1-truncated peptides (10 μg per lane) were separated by sodium dodecyl sulfate-polyacrylamide gel electrophoresis and visualized by staining with Coomassie brilliant blue. Molecular mass standard is shown to the left. (A-3) Sialidase activity of peptides. Recombinant peptide (50 μM) was incubated with 4-MU-Neu5Ac, and fluorescence was recorded every 15 min for 30 min. Data are presented as means ± standard deviations (*n* = 5). (C) Binding of sialidase peptides to PCM was evaluated by ELISA. Recombinant peptides (0.034 to 75 μM) were added to PCM-coated wells. Binding was detected with an anti-His antibody. Data are presented as means ± standard deviations (*n* = 3). Download FIG S6, TIF file, 1 MB. Copyright © 2017 Nishiyama et al. 2017 Nishiyama et al. This content is distributed under the terms of the Creative Commons Attribution 4.0 International license.

Microtiter plates coated with PCM were incubated with increasing concentrations of sialidase peptides, and binding was detected with an anti-His-tag antibody. His-SiaBb2 showed dose-dependent binding (Fig. 3D) that was equal to or greater than that of His-SiaBb1 (Fig. S6B). We next examined the role of the sialidase domain in mediating interactions with PCM. His-SiaBb2 and His-Sia showed dose-dependent binding to PCM, whereas His-ΔSia showed no binding (Fig. 3D). These results indicate that SiaBb2 can stably interact with PCM and that the sialidase domain (aa 187 to 553) is responsible for this interaction. To investigate the binding of His-SiaBb2 to the intestinal mucosa, we carried out binding assays using murine colonic mucosal tissue sections. Total acidic mucins were detected by alcian blue staining (Fig. 3E). Biotinylated His-SiaBb2 was found to specifically react with the mucosal surface at the border between the mucus layer and fecal materials as well as with goblet cells (Fig. 3E). The biotin control did not produce a signal (data not shown), indicating that SiaBb2 specifically interacts with colonic mucus.

To further dissect the role of enzymatic activity in mediating the interactions of SiaBb2, we used the sialidase inhibitor *N*-acetyl-2,3-didehydro-2-deoxyneuraminic acid (Neu5Ac2en). Neu5Ac2en treatment inhibited the sialidase activity of His-SiaBb2 in a dose-dependent manner, with maximal inhibition at 10 mM (Fig. S7A); it also affected the binding properties of His-SiaBb2 (Fig. S7B) and His-SiaBb1 (Fig. S7B). These results suggest that the sialidase domain is responsible for SiaBb2 binding, whereas enzymatic activity is less important.

10.1128/mBio.00928-17.8 FIG S7  Influence of sialidase inhibitor or Neu5Ac on the binding of SiaBb2 peptides to PCM. (A) Dose-dependent inhibition of His-SiaBb2 activity by Neu5Ac2en. (B and C) Binding of sialidase peptides to PCM in the presence of Neu5Ac2en (B) and Neu5Ac (C), as determined by ELISA. Binding was detected using an anti-His antibody. Data represent means ± standard deviations (*n* = 5). Differences were evaluated by analysis of variance with the Tukey test. *, *P* < 0.05 versus groups without Neu5Ac2en or Neu5Ac treatment. ns, nonsignificant. Download FIG S7, TIF file, 0.6 MB. Copyright © 2017 Nishiyama et al. 2017 Nishiyama et al. This content is distributed under the terms of the Creative Commons Attribution 4.0 International license.

### The sialidase domain binds to 6'SL and blood type A (BgA) antigen.

**FIG 4:**
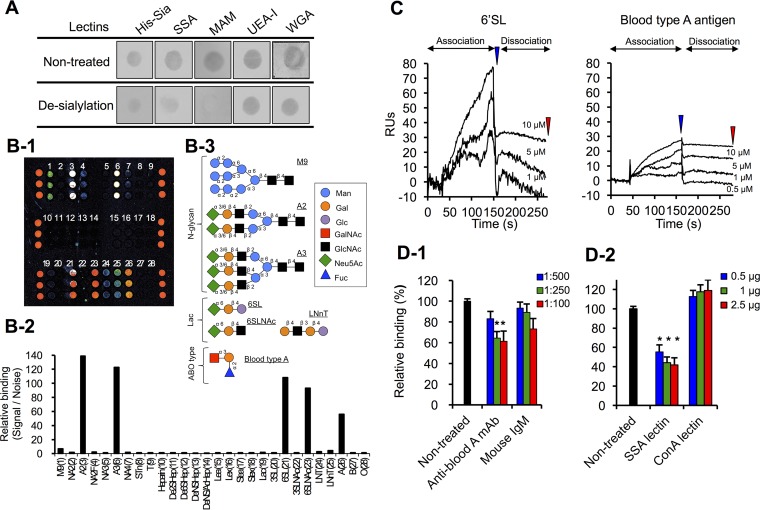
SiaBb2 binds to sialyl-(α2,6)Gal and BgA antigen. (A) Effects of desialylation with mild acid hydrolysis on binding of His-Sia and lectins to PCM. PCMs were immobilized on a nitrocellulose membrane. Binding of biotinylated His-Sia or lectins was detected with horseradish peroxidase-labeled streptavidin. SSA, *S. sieboldiana*; MAM, *M. amurensis*; UEA-I, *U. europaeus*; WGA, wheat germ agglutinin. (B) Glycoarray analysis. (B-1) Fluorescence intensity of His-Sia on the glycoarray plate measured with a fluorescence scanner. (B-2) Fluorescence signals presented as a histogram of signal/noise ratios calculated from three independent spots (fluorescence intensity of binding His-Sia signal divided by background noise); each bar represents a carbohydrate. (B-3) Schematic illustration of representative carbohydrate structures described in the study. (C) SPR sensorgrams of His-Sia interaction with 6'SL and BgA trisaccharide. Carbohydrates were added to a CM5 sensor chip with immobilized ligand His-Sia. Analyte concentrations (from top to bottom) are 10, 5, 1, and 0.5 μM. Blue and red arrowheads indicate the end of association and end of dissociation, respectively. (D) Inhibition of His-Sia binding to immobilized PCM by anti-BgA antibody (D-1) or sialyl-(α2,6)Gal-binding *S. sieboldiana* (SSA) lectin and mannose-binding ConA lectin (D-2). Binding of His-Sia to PCM, as determined by ELISA. Data represent means ± standard deviations (*n* = 5). Significant differences were determined by analysis of variance with a Tukey test. *, *P* < 0.05 versus His-Sia peptides preincubated without antibody (untreated) or lectins.

We investigated whether desialylation of PCM by mild acid hydrolysis would affect binding of recombinant sialidase domain peptides (i.e., His-Sia) by dot blot analysis. After desialylation, the reactivity of sialyl carbohydrate-binding lectins from *Sambucus sieboldiana* and *Maackia amurensis* was markedly reduced compared to binding to untreated PCMs, while binding to GlcNAc-binding lectin, wheat germ agglutinin, or fucose-binding lectin from *Ulex europaeus* I was unaffected (Fig. 4A). Although desialylation affected His-Sia binding, a signal was nonetheless observed (Fig. 4A). We examined whether Neu5Ac inhibits the binding of His-SiaBb2 to PCM and found that preincubation with Neu5Ac did not completely inhibit His-SiaBb2-binding (Fig. S7C). These results indicate that the sialidase domain recognizes carbohydrates other than the sialylated carbohydrates of PCM.

To explore the specificity of the sialidase domain for carbohydrates of mucin epitopes, we examined the interaction of His-Sia with 28 types of sugar chains using a glycoarray. Strong His-Sia fluorescence signals corresponding to carbohydrate chains of A2, A3, 6'SL, and 6'SLNAc—which have an α2,6 linkage at the nonreducing terminus—were detected (Fig. 4B-1 and -2). In contrast, His-Sia did not bind to SLe^a^, SLe^x^, 3′SL, and 3′SLNAc, which have an α2,3 linkage at the nonreducing terminus (Fig. 4B-1 and -2). These results indicate that the protein binds to the α2,6 rather than the α2,3 linkage. Interestingly, His-Sia moderately bound to BgA antigen [GalNAcα1-3(Fucα1-2)Galβ-]—which lacks sialic acid—but not to blood type O (BgO) [Fucα1-2(Galβ1-3)GlcNAcβ-] or blood type B (BgB) [Galα1-3(Fucα1-2)Galβ-] antigens (Fig. 4B-3).

In surface plasmon resonance (SPR) experiments using His-Sia-coupled biosensor chips, 6'SL and BgA trisaccharides bound rapidly and dose dependently to His-Sia (Fig. 4C). When 10 μM analyte was injected, the binding capacity—based on the molar ratio (MR) of analyte to ligand in the complex—of 6'SL (MR of 1.3) was higher than that of BgA (MR of 0.5) at the end of the association (Fig. 4C). In contrast, at the end of dissociation, binding levels were similar between 6'SL (MR of 0.5) and BgA (MR of 0.3) trisaccharides (Fig. 4C). These results indicate that SiaBb2 can interact with 6'SL and BgA trisaccharides, albeit with different affinities. In addition, pretreatment with Neu5Ac2en drastically decreased the binding of His-Sia to 6'SL (Fig. S8A) while having no effect on His-Sia binding to BgA trisaccharide (Fig. S8B). This suggests that although the binding of SiaBb2 to BgA antigen was substantially lower than that to the sialyl(α2,6)Gal residue at the nonreducing terminus of sugar chains, SiaBb2 may be able to recognize two different sugar chains of mucin.

10.1128/mBio.00928-17.9 FIG S8  (A and B) Influence of sialidase inhibitor on the binding of His-Sia peptides to 6'SL (A) or BgA trisaccharide (B). Surface plasmon resonance sensorgrams of His-Sia interaction with BgA trisaccharide or 6'SL in the presence of the sialidase inhibitor Neu5Ac2en (5 mM), which was added to HBS-EP running buffer. Carbohydrates (10 μM) were added to the CM5 sensor chip with immobilized ligand His-Sia. (C and D) Inhibition of *B. bifidum* ATCC 15696 adhesion to immobilized PCMs by anti-BgA antibody (C) or sialyl-(α2,6)Gal-binding *S. sieboldiana* lectin (D). PCM was preincubated with anti-BgA antibody (1:100 to 1:12.5 dilution) or *S. sieboldiana* lectin (0.5 to 2.0 μg), followed by addition of *B. bifidum* ATCC 15696 strain. Bound bacteria were stained with methylene blue, and randomly selected fields were imaged with a microscope. Data represent means ± standard deviations (*n* = 5). Differences were evaluated by analysis of variance with a Tukey test. *, *P* < 0.05 versus results without antibody or lectin treatment. Download FIG S8, TIF file, 0.9 MB. Copyright © 2017 Nishiyama et al. 2017 Nishiyama et al. This content is distributed under the terms of the Creative Commons Attribution 4.0 International license.

To investigate the contribution of the BgA antigen to mucin binding, we examined whether His-Sia binding to PCM could be inhibited by a monoclonal antibody (MAb) to BgA antigen or sialyl(α2,6)Gal residue-binding *S. sieboldiana* lectin. Pretreatment with anti-BgA MAb (Fig. 4D-1) or *S. sieboldiana* lectin (Fig. 4D-2) reduced the binding of His-Sia to PCM, while a mouse IgM control and mannose-binding lectin from *Canavalia ensiformis* (concanavalin A [ConA]) had little or no effect on the SiaBb2 signal. Thus, the conserved sialidase domain of SiaBb2 can bind to mucin via the sialyl(α2,6)Gal residue and BgA antigen on mucin. Moreover, the adhesion of *B. bifidum* ATCC 15696 to PCM was inhibited by anti-BgA MAb (Fig. S8C) and *S. sieboldiana* lectin (Fig. S8D), demonstrating that the binding of SiaBb2 to BgA antigen and the sialyl(α2,6)Gal residue promotes *B. bifidum* adhesion to the mucosal surface.

## DISCUSSION

**FIG 5:**
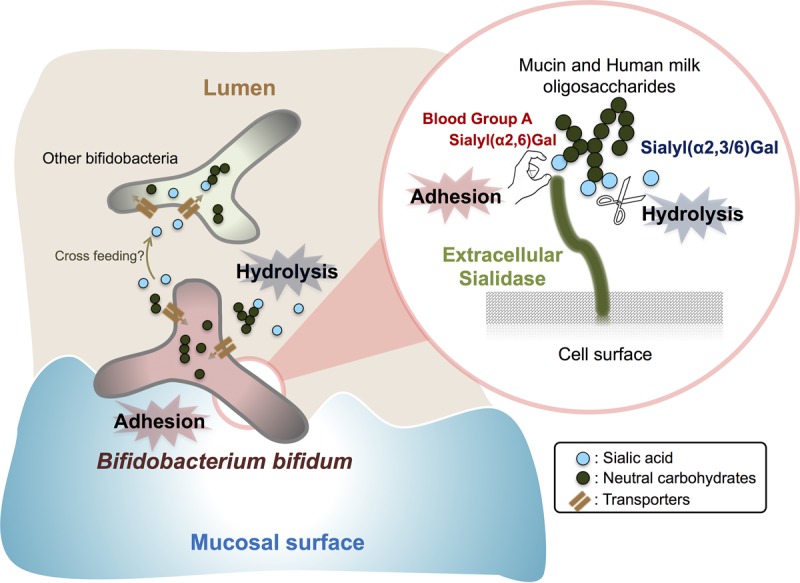
Novel functions of extracellular sialidase SiaBb2 in *B. bifidum* ATCC 15696. SiaBb2 enhances adhesion to the mucosal surface and supports assimilation of sialylated HMOs and intestinal mucin oligosaccharides by bifidobacteria.

Bifidobacteria are thought to employ a variety of mechanisms to facilitate colonization of the host GI tract (5, 11–15, 17). Here, we demonstrate that *B. bifidum* extracellular sialidase may modulate the assimilation of sialylated carbohydrates and *B. bifidum*-mucosal surface interactions based on the following observations. (i) SiaBb2 cleaved sialyl-HMO and -PMO to produce utilizable oligosaccharides, which supported the growth of *B. bifidum*. (ii) SiaBb2 promoted *B. bifidum* adhesion to the mucosa via specific binding to sialyl(α2,6)Gal residue and BgA antigen on mucin. These findings indicate that extracellular sialidase acts as a bifunctional enzyme that is potentially important for *B. bifidum* colonization (Fig. 5).

While several microbes have exosialidases for utilization of terminally sialylated glycans, the role of *B. bifidum* sialidase in HMO or mucin catabolism and utilization during growth remains largely unknown. In the present study, we demonstrated with a bacterial growth assay using a *siabb2* mutant *B. bifidum* ATCC 15696 strain that the cleavage of sialic acid from HMO and PMO by extracellular sialidase is important for growth. Interestingly, although there was little difference between wild-type and *Δsiabb2* strains in the early growth phase, disparities became apparent starting from the exponential growth phase, implying that since usable nutrient sources (i.e., nonsialylated carbohydrates) were depleted for both strains, wild-type cells gained a growth advantage after the mid-exponential growth phase owing to their extracellular sialidase activity. This is supported by the finding that when the two strains were cocultured with HMO as the sole carbon source, there was no difference in bacterial cell numbers starting from 24 h of culturing. Thus, *B. bifidum Δsiabb2* may depend on sialidase-expressing wild-type *B. bifidum* strains to acquire nonsialylated carbohydrates after mid-exponential growth phase. A recent study reported that *B. breve* harbors sialic acid metabolism genes (i.e., the Nan operon) but lacks extracellular sialidase and that cross-feeding occurred between sialic acid-releasing *B. bifidum* PRL2010 and sialic acid-utilizing *B. breve* UCC2003 (32). These results suggest that *B. bifidum* SiaBb2 stimulates the production of utilizable sialyloligosaccharides through a “selfish” function and supplies sialic acid or nonsialylated carbohydrates to other *Bifidobacterium* strains through an “altruistic” function (Fig. 5) (40).

We also investigated the role of SiaBb2 in the adhesion of *B. bifidum* ATCC 15696 to the mucosal surface. Adhesion assays with *Δsiabb2* and heterologous SiaBb2-expressing *B. longum* subsp. *longum* strains demonstrated that cell surface-associated SiaBb2 contributes to adhesion to mucins and mucus-secreting epithelial cells. Several proteinaceous adhesion factors have been identified in *B. bifidum* strains (11, 13–15); however, their functionality has been demonstrated only for Tad pili from *B. breve* UCC2003 (12). SiaBb1 and SiaBb2 peptides bound to PCM to similar degrees, while almost no adhesion was observed for the *Δsiabb2* mutant, which was correlated with the phenotypes of sialidase activity and sialyloligosaccharide utilization. Thus, SiaBb2 is the main determinant of the sialidase-associated phenotype of *B. bifidum* ATCC 15696 (i.e., carbohydrate assimilation and bacterial adhesion).

We showed that SiaBb2 is exposed on the bacterial surface in the early to late exponential growth phases but is buried within the peptidoglycan from the stationary phase onward. This suggests that since SiaBb2 is a membrane-bound protein, the peptidoglycan can mask SiaBb2 on the cell surface and sterically hinder SiaBb2 binding. This is in agreement with our finding that SiaBb2 functions as an adhesion factor in early to late exponential growth phase. Interestingly, after the stationary growth phase, *B. bifidum* ATCC 15696 still adhered to mucin, suggesting that after the early to late exponential growth phase, other factors promote bacterial adhesion and possibly enable *B. bifidum* colonization. Previous reports indicated that Fim and Tad pili are highly conserved across *Bifidobacterium* and that transcription of genes related to Fim and Tad pili is enhanced under certain conditions, e.g., in the colonization of mouse gut compared to *in vitro* cultures (12, 15). In our study, *B. bifidum* ATCC 15696 adhesion to PCM differed according to the carbon source (HMO and PMO), whereas the function of SiaBb2 as an adhesion factor was unaffected. Taken together, these data suggest that while SiaBb2-mediated adhesion is a strategy specific to *B. bifidum*, this species also has general adhesion factors such as pili (12, 15) that are used flexibly according to growth phase and environmental conditions. Therefore, further research is needed to determine whether *B. bifidum* SiaBb2 reaches the mucosal surface.

We demonstrated that the His-SiaBb2 peptide bound to PCM and murine colonic tissue, indicating that SiaBb2 can bind to the mucosal surface. Although the sialidase domain was essential for this process, addition of the transition-state analog Neu5Ac2en had no major effect, suggesting that cleavage and binding occur via independent mechanisms. This was unexpected, since most glycoside hydrolases possess carbohydrate-binding modules (CBMs) in addition to their catalytic domains that target enzymes to appropriate substrates to increase their catalytic efficiency. For example, *N*-acetyl-β-hexosaminidase from *Clostridium perfringens* containing family 32 CBMs mediates enzyme attachment to the terminal disaccharide β-d-galactosyl-1,4-β-d-*N*-acetylglucosamine motif common to *O*-linked glycans of mucin (41). *N*-Acetylglucosaminidase from *B. bifidum* JCM1254—containing four CBMs—interacts with mucin and the GH89 domain hydrolyzes mucin carbohydrates, suggesting that CBMs enhance affinity for porcine mucin but do not promote bacterial adhesion (42). Most bacterial sialidases, including SiaBb1, contain a CBM (ConA-like domain in this study) that acts as a stalk in the binding to carbohydrates such as sialic acid (43, 44) and galactose (45). However, the CBM of SiaBb2 is not conserved. We speculate that SiaBb2 containing a sialidase domain employs a novel mechanism for binding to carbohydrate moieties.

Glycoarray analysis showed that the sialidase domain binds to the α2,6 linkage of sialyloligosaccharide; however, there was no binding to the α2,3 linkage. Regarding the enzyme substrate specificity of SiaBb2, a previous study showed that cleavage efficiency is more than 20 times higher for the α2,3 than for the α2,6 linkage (34), suggesting that SiaBb2 preferentially binds the α2,6-linked residue. Surprisingly, the sialidase domain also bound BgA antigen. The different patterns of binding to sialyloligosaccharide and BgA antigen were evident in SPR sensorgrams, which showed rapid and strong binding to 6'SL initially, followed by a precipitous decline after dissociation. Conversely, although there was less binding to BgA antigen than to 6'SL, the binding properties were retained even after dissociation. These results suggest that SiaBb2 has distinct binding mechanisms for each of these carbohydrate chains. In support of this possibility, pretreatment with Neu5Ac2en did not affect the binding of His-Sia to BgA trisaccharide compared to sialyloligosaccharide, indicating that the BgA antigen-binding region(s) in the sialidase domain differs from the Neu5Ac-binding region (i.e., catalytic site). Thus, the cleavage-independent SiaBb2 binding to PCM described above may occur via interaction with the BgA antigen. In addition, since PCM desialylation and Neu5Ac addition had little effect on His-Sia binding, BgA antigen may be as important as the α2,6-linked residue for adhesion to the mucosal surface.

His-Sia exhibited specific binding to BgA antigen and did not bind to BgO and BgB antigens. Moreover, the interaction of His-Sia with PCM did not prevent the addition of monosaccharides such as GalNAc, fucose, and galactose in an enzyme-linked immunosorbent assay (ELISA) (data not shown). These findings suggest that SiaBb2 does not interact with monosaccharides and specifically recognizes complex sugar chains, which in turn implies that bifidobacterial sialidase has as-yet-undescribed (i.e., CBM-independent) binding properties. Blood group antigens are expressed in porcine intestinal mucin—with a predominance of BgA antigen (46)—as well as in human colonic mucin (47). Moreover, given that mucin glycosylation patterns vary markedly according to species, age, and location in the GI tract, SiaBb2-binding properties may likewise be affected. We therefore speculate that each of the carbohydrate chains (α2,6 linkage of sialyloligosaccharide and BgA antigen) dictates *B. bifidum* localization within the host GI tract and is a critical determinant of host specificity in *Bifidobacterium* strains. However, further analyses (e.g., bioinformatics) are necessary to clarify the SiaBb2-binding properties of BgA antigen.

The importance of bifidobacterial glycosidases and the mechanisms that enable the degradation and metabolism of HMO and mucin glycan have been widely reported. The results of this study provide new insights into the molecular mechanisms of nutrient acquisition and adhesion of *B. bifidum* ATCC 15696 involving the exo-α-sialidase SiaBb2. Although additional research is needed to determine whether SiaBb2-mediated adhesion is required for *Bifidobacterium* survival and colonization, we expect that other glycosidases expressed on the bacterial surface contribute to colonization of the mucosal surface.

## MATERIALS AND METHODS

See Text S1 in the supplemental material for full details.

10.1128/mBio.00928-17.1 TEXT S1  Supplemental materials and methods. Download TEXT S1, PDF file, 0.2 MB. Copyright © 2017 Nishiyama et al. 2017 Nishiyama et al. This content is distributed under the terms of the Creative Commons Attribution 4.0 International license.

### Bacterial strains and growth conditions.

*B. bifidum* ATCC 15696, *B. bifidum* JCM1254, and *B. longum* subsp. *longum* 105-A were anaerobically cultured at 37°C in de Man-Rogosa-Sharp (MRS; BD Difco, Le Pont de Claix, France) medium supplemented with 0.02% (wt/vol) l-cysteine and 0.34% (wt/vol) sodium ascorbate (MRSCS) or modified MRSCS (mMRSCS; without added sugar). Half-strength MRS medium (1/2 MRSCS) was used for gene disruption and complementation analyses. Chloramphenicol (Cm) and spectinomycin (Sp) were added at various concentrations as required. *Escherichia coli* strains DH5α and BL21(DE3) were grown in Luria-Bertani broth at 37°C, and when necessary, ampicillin, kanamycin, and Sp were added at 100, 50, and 100 μg/ml, respectively.

### Bifidobacterial growth assay.

HMOs from human milk were prepared as previously described by chloroform-methanol extraction and gel filtration chromatography (48). PMOs from PCM (isolated from 24-week-old Landrace swine) were prepared as previously described by sodium borohydride treatment and gel filtration chromatography (49). The bacterial growth assay was carried out according to a published protocol (48), with several modifications. mMRSCS medium was supplemented with a 1% (wt/vol) carbon source (lactose [Wako, Tokyo, Japan], 6'SL [Carbosynth Limited, Berkshire, United Kingdom], HMOs, or PMOs). An 0.3-ml volume of bacterial suspension (equivalent to an OD_600_ of 0.1) was then added to the medium (3 ml) followed by incubation under anaerobic conditions at 37°C. Bacterial cell concentration was determined by measuring OD_660_ on a MiniPhoto 518R spectrophotometer (Taitech Co., Tokyo, Japan).

For the cogrowth assay, *B. bifidum* ATCC 15696 pKKT427-Cm^r^ (in which the empty vector pKKT427-Cm^r^ was transformed into the wild-type strain as a selection marker) and the *Δsiabb2* (Sp^r^) strain were inoculated into 1% (wt/vol) HMO-supplemented mMRSCS medium at a 1:1 ratio, corresponding to a final OD_660_ of approximately 0.1. Collected samples were serially diluted in modified Mitsuoka’s buffer (50). The number of CFU of each strain was determined by counting colonies on 1/2 MRSCS agar plates containing Cm (2.25 μg/ml) for *B. bifidum* ATCC 15696 pKKT427-Cm^r^ or Sp (75 μg/ml) for the wild-type strain. Cells were cultured under anaerobic conditions at 37°C for 48 h.

### Bacterial adhesion assay.

*B. bifidum* ATCC 15696 strains (wild type, Δ*siabb2*, and Δ*siabb2*+p*siabb2*), *B. bifidum* JCM1254, and *B. longum* subsp. *longum* 105-A were anaerobically cultured in MRSCS medium at 37°C for 24 h. *Bifidobacterium* strains were diluted 1:100 in fresh MRSCS or 1% (wt/vol) HMO- or PMO-supplemented mMRSCS medium and cultured at 37°C for indicated periods (12 h for *B. longum* subsp. *longum* 105-A strains). Cells were harvested by centrifugation (6,000 × *g*, 5 min, 4°C) and resuspended in Dulbecco’s modified Eagle’s medium (DMEM).

Bacterial adhesion to PCM was assessed according to a published method (17), with several modifications. Diagnostic glass slides (Matsunami Glass Industry, Osaka, Japan) were coated with 40 μl purified PCM (1 mg/ml) for 12 h at 4°C. After washing three times with phosphate-buffered saline (PBS) containing 0.05% (wt/vol) bovine serum albumin (BSA), slides were incubated with 2% (wt/vol) BSA-PBS for 1 h at room temperature. A 40-μl volume of bacterial suspension (equivalent to an OD_600_ of 0.5) was added to the wells followed by incubation at 25°C for 2 h. Samples were washed three times with 0.05% (wt/vol) BSA-PBS to remove unbound bacteria and stained with methylene blue. Randomly selected fields were imaged with an AX10 microscope (Carl Zeiss, Inc., Tokyo, Japan), and bacteria in five fields were counted.

Bacterial adhesion to epithelial cells was assayed as previously described (51), with several modifications. Bacterial cells were resuspended in DMEM at a ratio of interaction of 1:500 and added to a confluent monolayer of epithelial cells (~2 × 10^5^ cells/well) followed by incubation at 37°C for 1 h. After two washes with PBS, cells were harvested with a cell scraper and resuspended in modified Mitsuoka’s buffer (50). To count adherent bacteria, serial dilutions of bacteria were plated on 1/2 MRSCS agar (with Sp [75 μg/ml] added when necessary) and cultured under anaerobic conditions at 37°C for 48 h. Relative adhesion was calculated as a percentage from five independent experiments with the following formula: 100 × (number of adherent bacteria/number of bacteria inoculated).

To test the inhibition of adhesion by anti-SiaBb2 antibody (see Text S1), bacterial cells were incubated with the antibody or with rabbit preimmune serum (1:100 dilution) under anaerobic conditions at 37°C for 1 h, washed twice with DMEM, and added to PCM-coated wells or epithelial cell monolayers. Bacterial adhesion was analyzed as described above.

To test the inhibition of adhesion by anti-BgA MAb or *S. sieboldiana* lectin, anti-BgA MAb (1:100, 1:25, and 1:12.5 dilutions; GeneTex, Irvine, CA) or *S. sieboldiana* lectin (0.5, 1, and 2.0 μg; J-Oil Mills, Tokyo, Japan) was added to PCM-coated wells followed by incubation for 30 min. After three washes with 0.05% BSA-PBS, bacterial adhesion was analyzed as described above.

### ELISA.

A 96-well microplate was coated with PCM (0.1 mg/well) and blocked with 2% (wt/vol) BSA-PBS for 1 h at room temperature. After two washes with 0.05% (wt/vol) BSA-PBS, His-tagged recombinant proteins were added to the wells, followed by incubation for 1 h at room temperature. After washing, proteins were detected with biotinylated anti-6×His-tag IgG (Novagen, Madison, WI; 1:1,500 dilution) followed by horseradish peroxidase-labeled streptavidin (Abcam, Inc., Cambridge, MA; 1:5,000 dilution). Tetramethylbenzidine (TMB) peroxidase substrate (KPL, Gaithersburg, MD) was added, and the reaction was terminated with 1 M phosphoric acid. Absorbance at 450 nm was measured on a SpectraMax M5 plate reader.

To evaluate the effect of neuraminidase inhibitor or neuraminic acid, proteins were incubated with Neu5Ac2en (1 or 10 mM; Nacalai Tesque, Kyoto, Japan) or Neu5Ac (1 or 10 mM; Nacalai Tesque) for 30 min and then added to a PCM-coated plate. Protein binding was analyzed as described above.

To determine whether antibody or lectins reduced His-Sia binding to PCM, anti-BgA MAb (1:100, 1:250, and 1:500 dilutions) or *S. sieboldiana* lectin (0.5, 1, and 2.5 μg) was added to PCM-coated wells followed by incubation for 30 min. After three washes with 0.05% BSA-PBS, adhesion was assayed as described above.

### Glycoarray analysis.

Biotinylated His-Sia (20 µg) was resuspended in 50 mM Tris-HCl (pH 7.5) containing 100 mM NaCl, 2 mM CaCl_2_, 2 mM MnCl_2_, 2 mM MgCl_2_, and 0.05% (vol/vol) Tween 20. The mixture was added to a glycoarray plate (Sumitomo Bakelite, Tokyo, Japan) followed by incubation for 2 h at room temperature. After washing, Cy3-conjugated streptavidin (1:1,000 dilution; Thermo Fisher Scientific, Waltham, MA) was added, and the plate was incubated for 60 min. Bound His-Sia was measured with a ScanArray Lite fluorescence confocal scanner (GSI Lumonics, Billerica, MA).

### SPR analysis.

The interaction of His-Sia with 6'SL and BgA trisaccharides [GalNAc-α-1,3-(Fuc-α-1,2)Gal; Carbosynth, San Diego, CA] was assessed by SPR using a Biacore X-100 instrument (GE Healthcare, Milwaukee, WI). His-Sia (1,290 resonance units [RU]) was immobilized on a CM5 (flow cell 2) dextran sensor chip (GE Healthcare); flow cell 1 was used as a blank. Experiments were performed at a flow rate of 20 µl/min in HBS-EP buffer (10 mM HEPES, 150 mM NaCl, 3 mM EDTA, and 0.005% Tween 20 [pH 7.4]) or 5 mM Neu5Ac2en in HBS-EP buffer at 25°C. Dissociation was carried out at the same flow rate for 120 s, 6'SL and BgA trisaccharides were injected, and RU was measured at the start of dissociation without further sample addition; signals were corrected for nonspecific binding by subtracting the blank. MRs based on the relative mass of analytes and ligands are presented for each sensorgram.

### Statistical analysis.

Data were analyzed using Prism 6 software (GraphPad Inc., La Jolla, CA). Statistical tests used to analyze each set of data are indicated in the figure legends; *n* represents the number of individual experiments. A *P* value of <0.05 was considered significant.

### Ethics statement.

All animal work was carried out in accordance with the guidelines for the care and use of laboratory animals of the School of Veterinary Medicine, Kitasato University, Japan.

10.1128/mBio.00928-17.10 TABLE S1  Primers used in this study. Download TABLE S1, PDF file, 0.1 MB. Copyright © 2017 Nishiyama et al. 2017 Nishiyama et al. This content is distributed under the terms of the Creative Commons Attribution 4.0 International license.
