# Supplementary figures and images for: Bifidobacterium bifidum Extracellular Sialidase Enhances Adhesion to the Mucosal Surface and Supports Carbohydrate Assimilation
Source: mBio. 2017 Oct 3;8(5):e00928-17. doi: 10.1128/mBio.00928-17 (PMC5626965; doi:10.1128/mBio.00928-17)

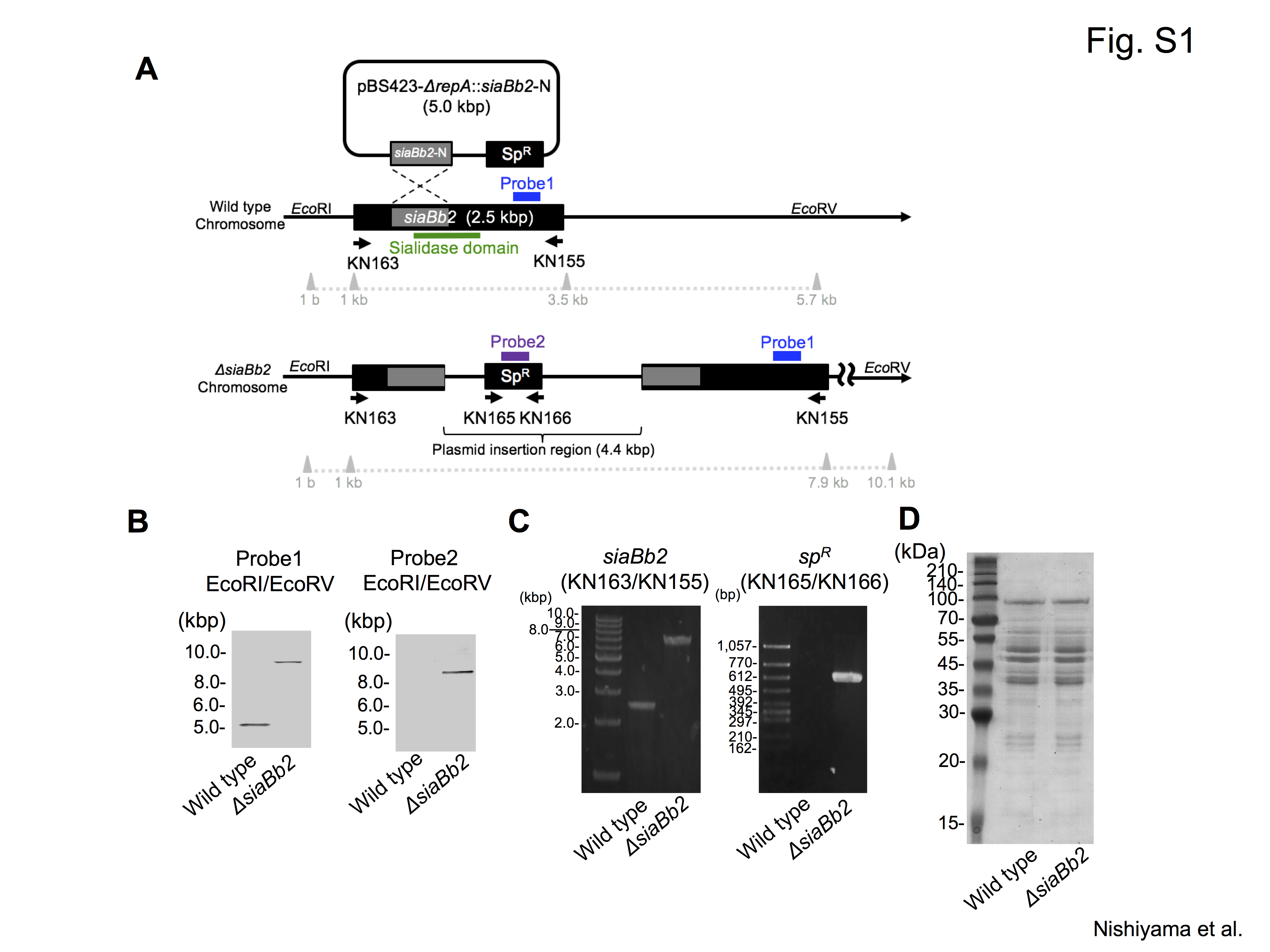

Supplement: FIG S1 [file mbo005173503sf1.tif]

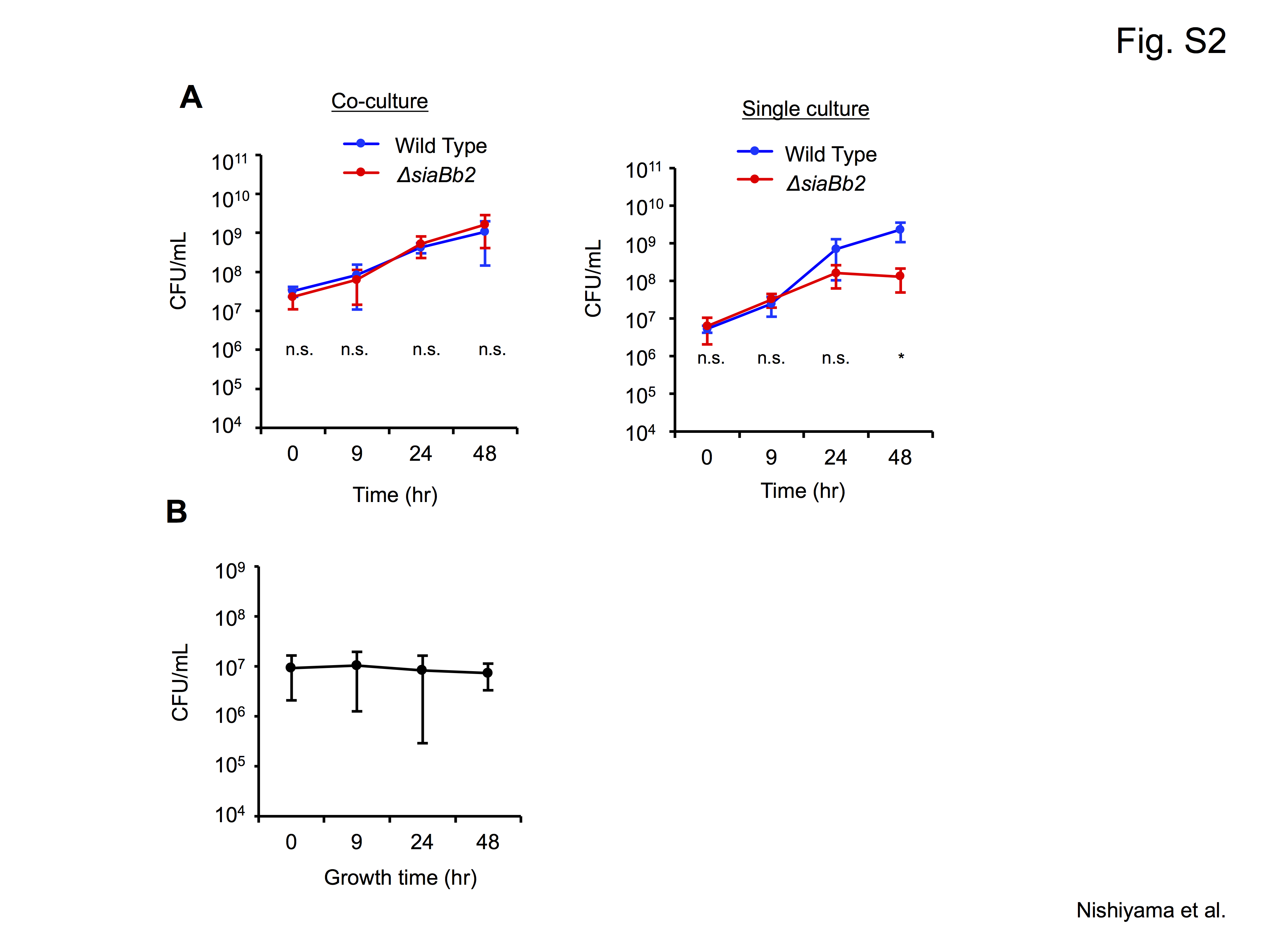

Supplement: FIG S2 [file mbo005173503sf2.tif]

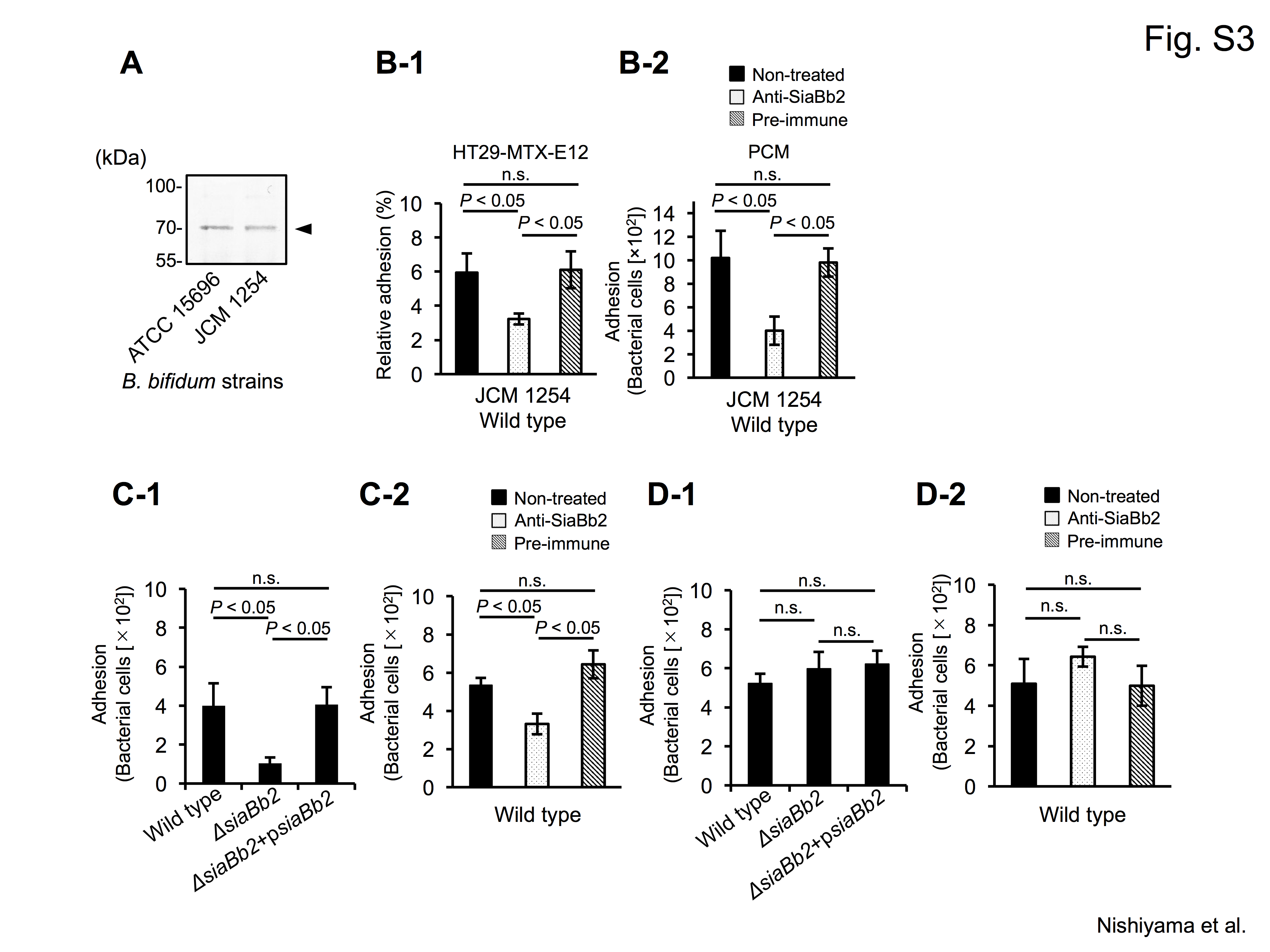

Supplement: FIG S3 [file mbo005173503sf3.tif]

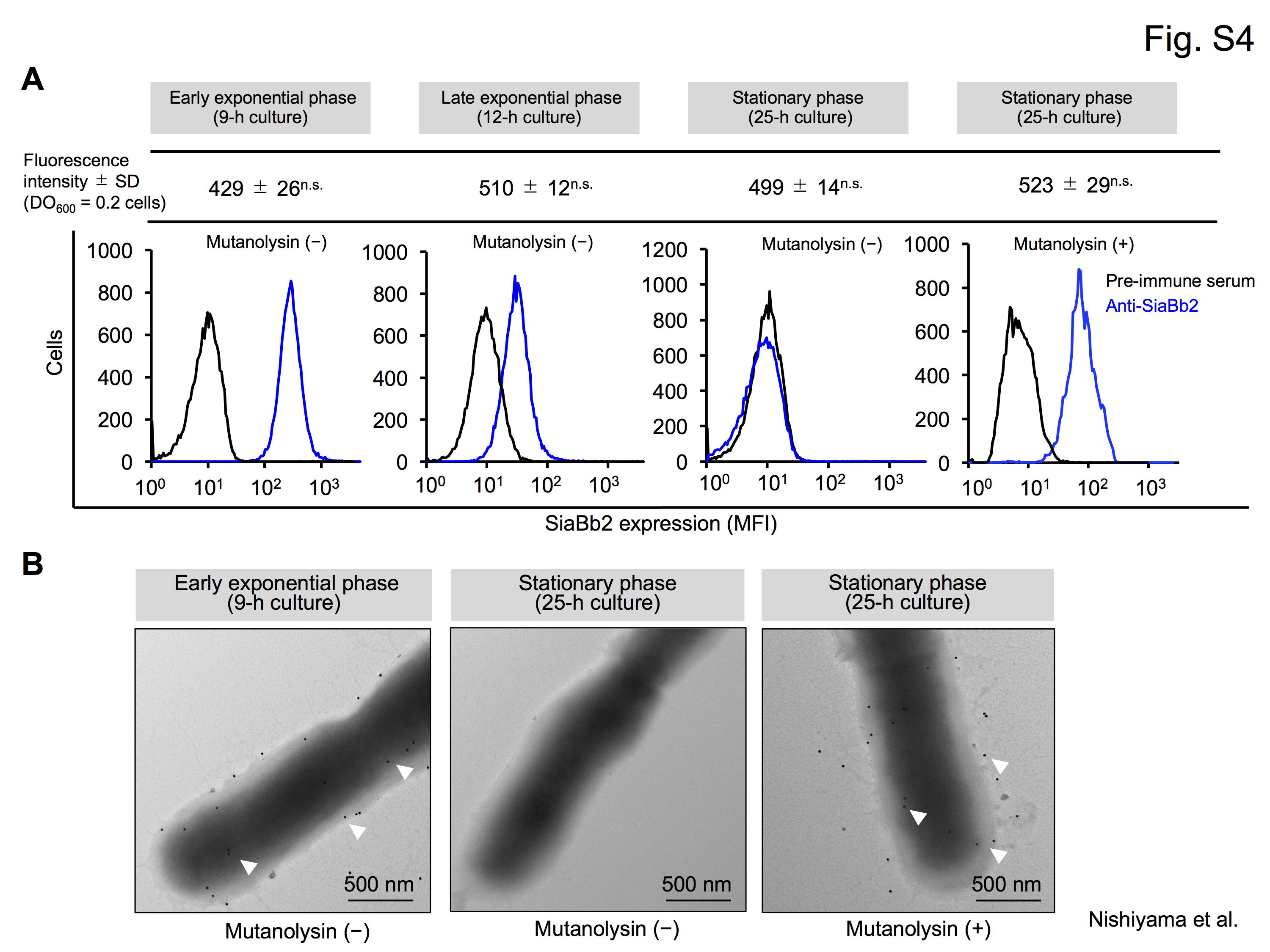

Supplement: FIG S4 [file mbo005173503sf4.tif]

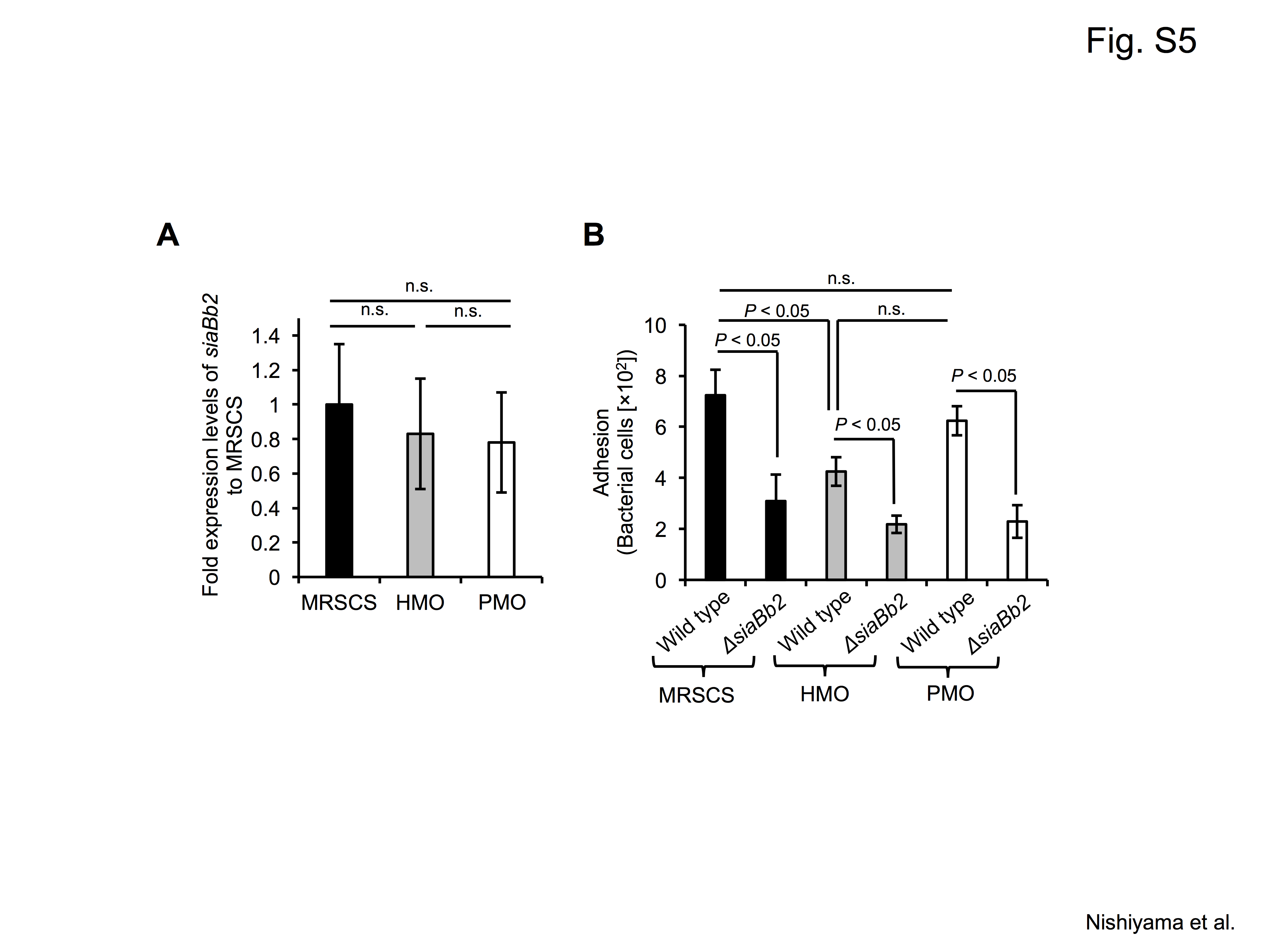

Supplement: FIG S5 [file mbo005173503sf5.tif]

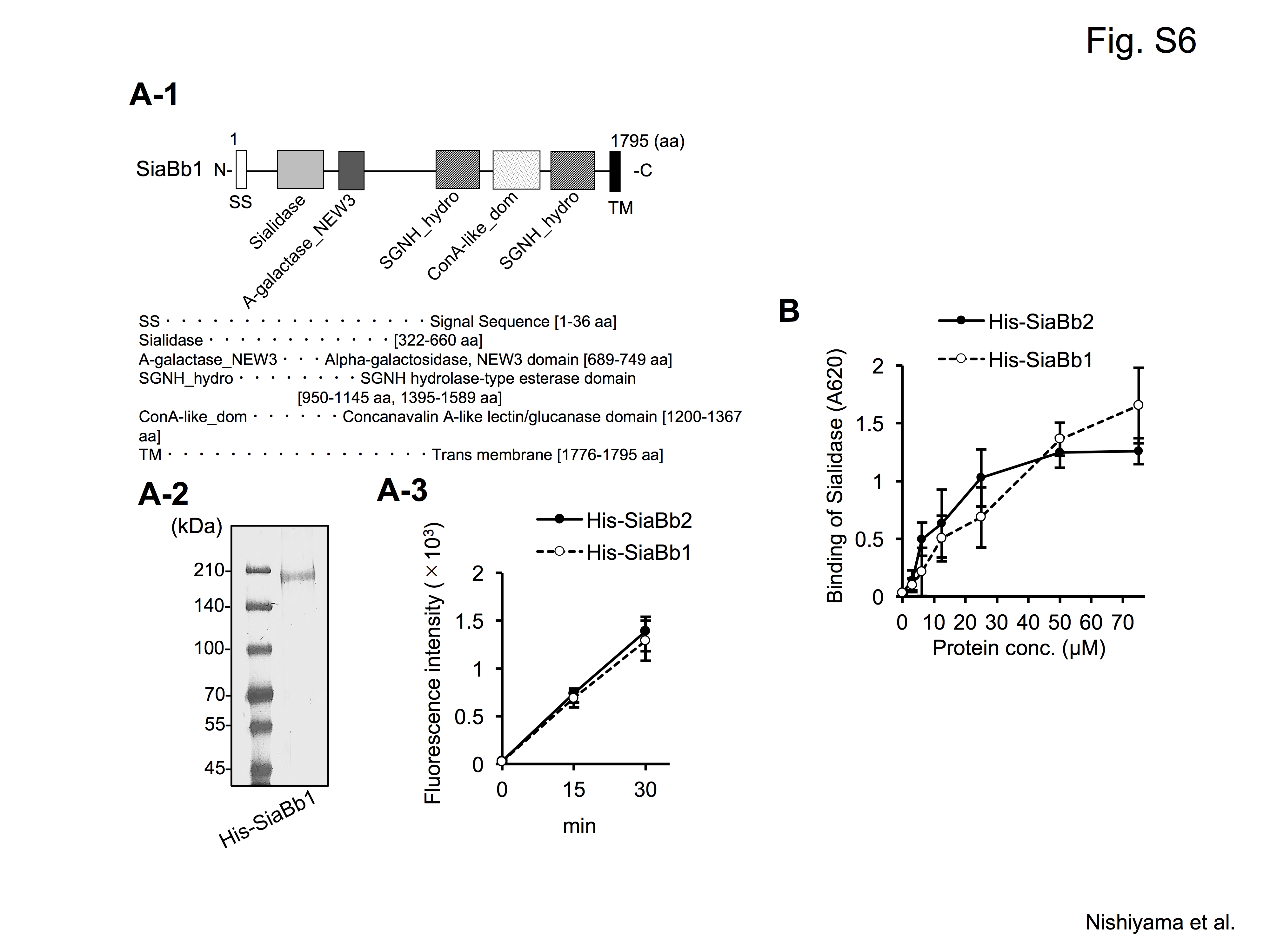

Supplement: FIG S6 [file mbo005173503sf6.tif]

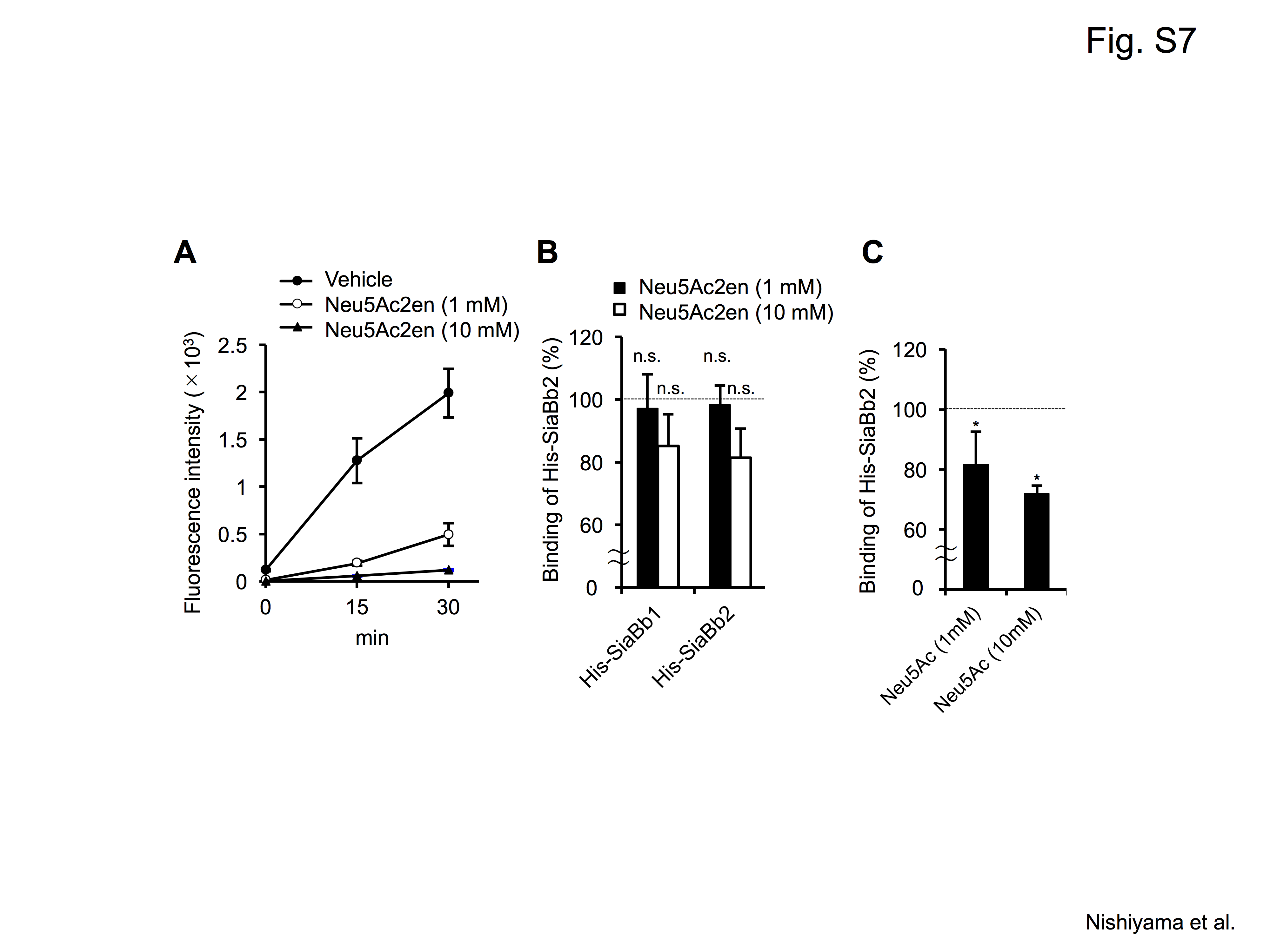

Supplement: FIG S7 [file mbo005173503sf7.tif]

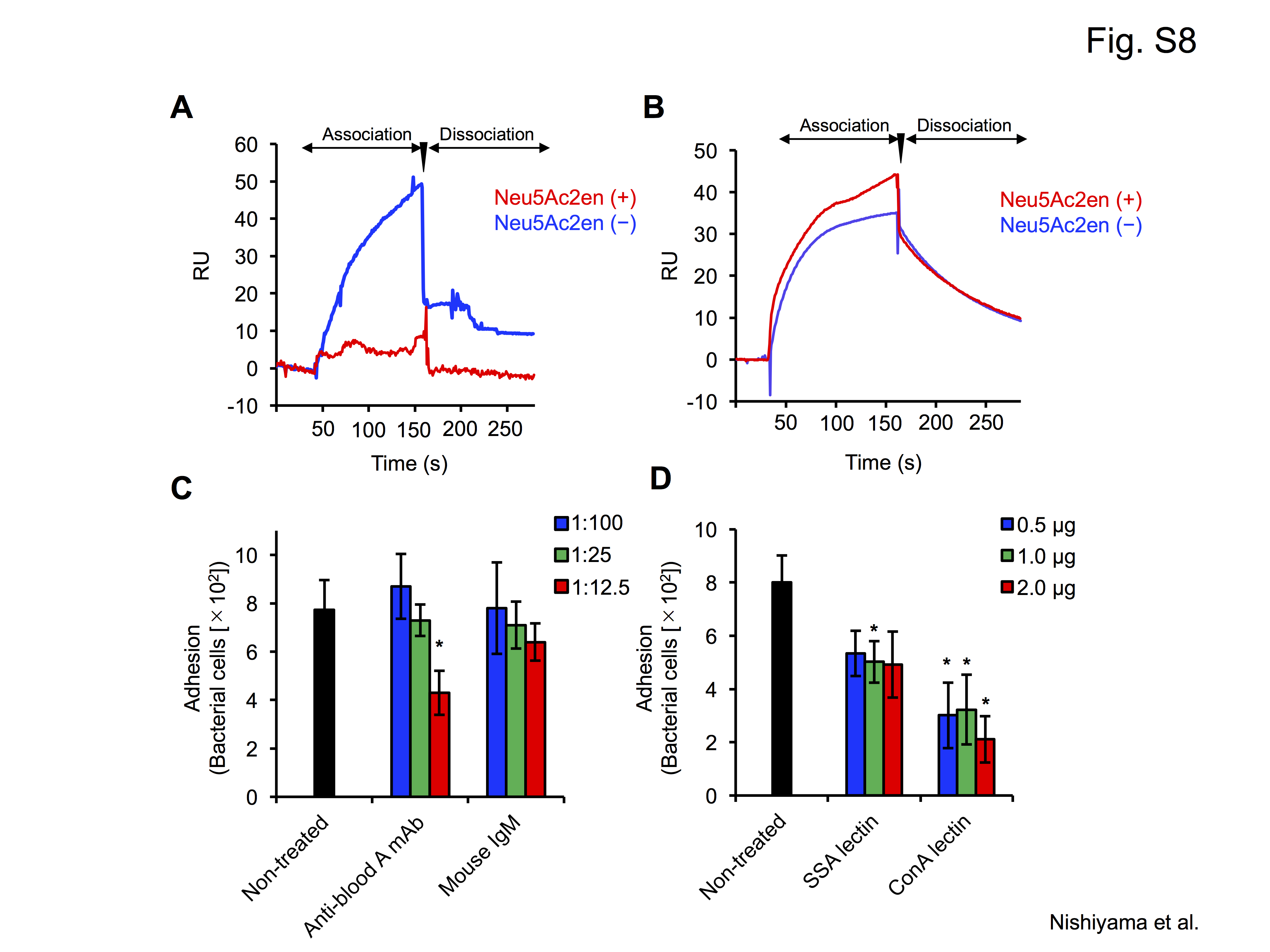

Supplement: FIG S8 [file mbo005173503sf8.tif]
