# Supplementary material for: Bifidobacterium bifidum Extracellular Sialidase Enhances Adhesion to the Mucosal Surface and Supports Carbohydrate Assimilation
Source: mBio. 2017 Oct 3;8(5):e00928-17. doi: 10.1128/mBio.00928-17 (PMC5626965; doi:10.1128/mBio.00928-17)
Supplement: TEXT S1 [file mbo005173503s1.pdf]

Bifidobacterium bifidum extracellular sialidase enhances adhesion to the mucosal surface and supports carbohydrate assimilation

Keita Nishiyama, Yuji Yamamoto, Makoto Sugiyama, Takashi Takaki, Tadasu Urashima, Satoru Fukiya, Atsushi Yokota, Nobuhiko Okada, and Takao Mukai

Text S1, SUPPLEMENTAL MATERIALS AND METHODS

Abbreviations:

BgA: Blood type A; HMO: Human milk oligosaccharide; MAM: *Maackia amurensis*; Neu5Ac: *N*-acetylneuraminic acid; Neu5Ac2en: *N*-acetyl-2,3-didehydro-2-deoxyneuraminic acid; PCM: porcine colonic mucin; PMO: Porcine mucin oligosaccharide; SPR: Surface plasmon resonance; SSA: *Sambucus sieboldiana*; UEA-I: *Ulex europaeus* I; WGA: wheat germ agglutinin; 4-MU-Neu5Ac: 2'-(4-methylumbelliferyl)-α-D-*N*-acetylneuraminic acid; 6'SL: 6'sialyl-lactose.

Epithelial cell line and culture conditions. Human intestinal epithelial mucous-secreting HT29-MTX-E12 cells (originated from HT29 and selected by growth adaptation to methotrexate, obtained from ECACC) were cultured in Dulbecco's modified Eagle medium (DMEM) supplemented with 10% (v/v) fetal bovine serum (FBS), 10 U/mL penicillin, and 10 μg/mL streptomycin at 37°C, 5% CO_2. Cells were grown for 21 days, and the medium was changed every 2 days. For adhesion and infection assays, cells were seeded in antibiotic- and FBS-free medium 24 h before the experiments.

Electrotransformation of *Bifidobacterium* strains. Electrotransformation of *Bifidobacterium* strains was performed using a previously described with several modifications (1). *B. bifidum* ATCC15696 was cultured anaerobically in 1/2 MRSCS medium at 37°C for 12 h. Cells were then diluted 1:50 in fresh 1/2 MRSCS medium supplemented with 0.5 M sucrose cultured until the OD_{600} reached 0.2. Cells were harvested by centrifugation at 8,000 × *g* at 4°C for 10 min and washed twice with ice-cold sucrose solution

(0.5 M sucrose, 10% [v/v] glycerol) and were re-suspended in about 1/300 of the original culture volume of ice-cold buffered sucrose (7 mM potassium phosphate buffer containing 0.5 M sucrose and 1 mM $MgCl_2$, pH 7.4). Electroporation was conducted using Gene Pulser (Bio-Rad laboratories, Hercules, CA, USA) with settings of 12 kV/cm, 25 μF, and 200 Ω. After an electric pulse, the cells were cultured anaerobically in 1/2 MRSCS supplemented with 0.5 M sucrose for 3 h at 30°C and spread onto 1/2 MRSCS agar. Usually high-frequency competent cells (approx. 1×10^5 CFU Sp-resistant transformants per μg of pKKT427 plasmid DNA (2)) was used for target gene disruption analysis.

Targeted gene disruption and complementation analysis. The schematic representation of genomic structure of *Bifidobacterium bifidum* ATCC 15696 *siaBb2* gene (GenBank: LC228603), the targeting vector, and the resultant mutant generated by homologous recombination was indicated in Fig. S1A. The N-terminal 0.65-kbp region of *SiaBb2* gene was amplified by PCR using Tks Gflex DNA Polymerase (Takara Shuzo, Shiga, Japan) using genomic DNA as a template and the primer pairs KN149/KN150 (Table S1). The amplified fragment was ligated into the *Hin*dIII fragment of pBS423-*ΔrepA* (pTB4 *ori ΔrepA* Sp^R) (2) to generate a suicide plasmid, designated as pBS423-*ΔrepA*::*siaBb2*-N. The resulting plasmid was introduced into *B. bifidum* ATCC 15696 by electroporation. Selection was performed on 1/2MRSCS agar plates containing 75 μg/mL spectinomycin (Sp) at 37°C. As a second selection, Sp-resistant colonies were evaluated for sialidase activity using 2'-(4-methylumbelliferyl)-α-D-*N*-acetylneuraminic acid (4-MU-Neu5Ac, refer to “Sialidase activity assay”). An Sp-resistant and enzyme-inactive colony was selected, and a single cross-over event at the *siaBb2* locus was confirmed by southern hybridization analysis (Fig. S1-B), PCR with the primer pair KN163/KN155 (for *siaBb2* gene) and KN165/KN166 (for Sp^R gene) (Table S1, Fig. S1C), and DNA sequencing.

For southern hybridization analysis, *Eco*RI and *Eco*RV-digested chromosomal DNA (5 μg) from each strain was subjected to 0.7 % agarose gel electrophoresis and the separated DNA bands were capillary blotted to a nitrocellulose membrane. The internal regions of *siaBb2* (0.35 kb) and Sp^R (0.5 kb) genes were amplified from *B. bifidum* ATCC15696 genomic DNA with the primer pair KN394/KN395 and KN396/KN397 (Table S1) using digoxigenin (DIG)-labeling kit (Roche, Indianapolis, IN, USA). Signal detection and visualization were using alkaline phosphatase (AP) conjugated DIG antibody (1:2,000 dilutions, Roche) and BCIP/NBT liquid substrate system (Nacalai Tesque, Kyoto, Japan).

Complementation analysis was performed using *Escherichia coli*-Bifidobacteria

shuttle plasmid pKKT427 (ColE1 *ori* pTB6 *ori* Sp^R) (3). pTBR101-CM (pMB1 *ori* pTB4 *ori* Cm^R, Ap^R) plasmid (2) was digested with *Hind*III to obtain a Cm^R cassette, which was ligated into *Hind*III-digested pKKT427, and the resulting plasmid was named pKKT427-Cm^R. The *siaBb2* gene containing the 0.24-kbp upstream regions was amplified by PCR using primers KN154/KN155 (Table S1), digested with *Hin*dIII, and cloned into pKKT427-Cm^R, then designated as pKKT427-Cm^R-*siaBb2*. The plasmid was transformed into the *B. bifidum* ATCC 15696 *siaBb2* mutant *ΔsiaBb2*, and selection was performed on 1/2MRSCS agar plates containing 2.25 μg/mL chloramphenicol.

To examine the physiological functions of SiaBb2 in hetero bifidobacteria, we expressed *siaBb2* from *B. bifidum* ATCC 15696 in *Bifidobacterium longum* 105-A, which does not have sialidase activity (4). The plasmid pKKT427-Cm^R-*siaBb2* was transformed into wild-type *B. longum* 105-A, and selection was performed on 1/2MRSCS agar plates containing 75 μg/mL Sp.

Expression and purification of recombinant proteins. Signal peptides and transmembrane domains were predicted using SignalP 4.1 (http://www.cbs.dtu.dk/services/SignalP/) and TMHMM 2.0 (http://www.cbs.dtu.dk/services/TMHMM/), respectively; predicted domains were obtained using InterPro (http://www.ebi.ac.uk/interpro/). Recombinant truncated sialidase proteins were expressed in *E. coli* and purified using N-terminal His-tags. *B. bifidum* ATCC 15696 sialidase genes coding for SiaBb1 (reference gene sequence from *B. bifidum* JCM 1254, GenBank: BAN14906.1) (primers KN314/KN315), SiaBb2 (primers KN310/KN311), and the Sia domain (aa 232−524, Pfam: PF13859) of SiaBb2 (primers KN318/KN319) were amplified by PCR with Tks Gflex DNA Polymerase using genomic DNA as a template and the primer pairs listed in Table S1. The amplified fragments were inserted into pET28b (Novagen, Madison, WI, USA) at indicated restriction sites (Table S1), then designated as pET28b::*siaBb1*, pET28b::*siaBb2*, and pET28b::*sia*, respectively (Fig. 3A-2). To eliminate the sialidase domain, inverse PCR using primers KN325/326 followed by blunt-end ligation was conducted using pET28b::*siaBb2* (Table S1), which was then designated as pET28b::*Δsia*. All resulting plasmids were confirmed by sequencing, and all plasmid DNA was introduced into *E. coli* BL21 (DE3).

Transformed cells were grown in LB medium at 30°C with shaking. When the OD_{600} reached 0.4, isopropyl-β-D-thiogalactopyranoside (0.2 mM) was added to induce protein expression. Bacteria were lysed with BugBuster Protein Extraction Reagent (Novagen) containing benzonase (Sigma-Aldrich, Tokyo, Japan) and lysozyme. Soluble proteins were

purified over a TALON metal affinity resin (Clontech Laboratories, Inc., Palo Alto, CA, USA) and Capto DEAE (GE Healthcare, Milwaukee, WI, USA) according to the manufacturers' manuals. Purified proteins were dialyzed against 20 mM HEPES buffer (pH 7.0). Protein concentrations were determined with a BCA protein assay kit (Takara Shuzo).

Preparation of *B. bifidum* whole cell extracts and western blotting. Bacterial cells were cultivated anaerobically in 5 mL MRSCS broth at 37°C. Bacteria were harvested by centrifugation (8,000 ×*g*, 5 min, 4°C), suspended in 0.5 mL of 50 mM Tris-HCl (pH 8.0) containing proteinase inhibitor (Roche), and subjected to bead beating for 180 s at 4,800 rpm using 0.3 g of 0.1-mm zirconia-silica beads (Bio Spec Products, Bartlesville, OK, USA). Cell debris was removed by centrifugation at 16,000 × *g*, 4°C, for 1 min. The protein concentration was determined using a BCA protein assay kit.

Antisera against the SiaBb2 synthetic peptide "ASDDADMLTVTMTRTDALGC" were raised in rabbits by routine immunization procedures. Protein samples (10 μg) were separated by 10% SDS-PAGE and transferred to a polyvinylidene difluoride membrane. The membrane was blocked with 5% (w/v) skim milk in phosphate-buffered saline (PBS)-0.05% (v/v) Tween-20 (PBS-T) for 15 h at 4°C. After washing the membrane with PBS-T, it was incubated with rabbit anti-SiaBb2 antibody diluted 1:500 in PBS-T at room temperature for 1 h. The membrane was washed and incubated with AP-conjugated goat anti rabbit IgG (Sigma-Aldrich) diluted 1:2,000 in PBS-T at room temperature for 1 h. After washing, the signals were developed with a BCIP/NBT liquid substrate system.

Detection of bacterial cell-surface SiaBb2 by flow cytometry. Flow-cytometric analysis was carried out as previously described (5). Briefly, bacterial cells were grown in MRSCS broth at 37°C for 9 or 25 h. The cells were washed with PBS and then suspended in 3 mL of PBS at approx. 10^8 CFU/mL. For enzymatic treatment, cells were suspended in mutanolysin buffer (50 mM Tris-HCl buffer [pH 7.0], 34% [w/v] sucrose, and 20 U mutanolysin [Sigma-Aldrich]) and incubated for 1 h at 37°C. The cells were incubated with rabbit anti-SiaBb2 antibody (1:400 dilution) for 1 h on ice. Then, the cells were incubated with goat anti-rabbit IgG conjugated with fluorescein isothiocyanate (Wako, Tokyo, Japan, 1:1,500 dilution) for 1 h in the dark. The samples were meshed and analyzed on a MoFlo XDP cell sorter (Beckman Coulter, Inc., Brea, CA, USA).

Immunoelectron microscopy. Bacterial cells were grown in MRSCS broth at 37°C for 9 or

25 h. The cells were centrifuged at 3,000 × *g* for 5 min and washed twice with PBS. For enzymatic treatment, cells were suspended in mutanolysin buffer and incubated for 1 h at 37°C. Following this step, cells were fixed with 4% paraformaldehyde and blocked with PBS containing 0.1% (w/v) gelatin for 30 min. After then, cells were washed twice and stained with rabbit anti-SiaBb2 antibody (1:300 dilution) for 2 h. Cell were washed twice and staining with 15-nm gold particles goat anti-rabbit IgG (BBI solutions, Cardiff, UK) (1:20 dilution) in PBS with 0.01% gelatin for 1 h. A drop of bacterial suspension was placed onto nickel-coated carbon grids (Nisshin EM Co., Ltd., Tokyo, Japan) and viewed with a transmission electron microscope H-7600 (Hitachi Co. Ltd., Japan).

Sialidase activity assay. A quantitative assay utilizing 4MU-Neu5Ac (Sigma-Aldrich) was used to assay sialidase activity. Recombinant protein (50 μM) was mixed with 50 μL of enzyme reaction buffer (50 mM sodium acetate, 150 mM NaCl [pH 5.0], 200 μM 4MU-Neu5Ac). Enzyme reactions were carried out at 37°C and stopped by the addition of glycine buffer (pH 10.7). Free 4-MU was determined based on fluorescence emission at 450 nm (excitation at 360 nm) on a SpectraMax M5 plate reader (Molecular Devices, Sunnyvale, CA, USA).

Sialidase activities of bifidobacterial cells were examined as described previously (4) with several modifications. Briefly, *Bifidobacterium* strains were grown in MRSCS broth for 15 h. The cells were washed PBS once, and ten mg (wet weight) of bacterial cells was suspended in 200 μL of enzyme reaction buffer. When screening *siaBb2* gene mutant (see "Targeted gene disruption and complementation analysis"), 200 μL of bacterial culture were pelleted and then equal volume of enzyme reaction buffer were added. After incubation, an aliquot of the supernatant was mixed with an equal volume of glycine buffer to quantify liberated 4MU.

Gene expression analysis. Nucleotide sequences of *siaBb2* gene from *B. bifidum* ATCC 15696 were used to design specific primers for real-time reverse transcription PCR (qRT-PCR) using primer-BLAST at NCBI (*siaBb2* primers: KNq392 and KNq393; Table S1). For relative quantification, *uvrD-rep* was used (6) (Table S1). *B. bifidum* ATCC 15696 was grown in mMRSCS medium supplemented with either 1% HMO, or 1% PMO, and cultures were sampled at the early exponential phase (HMO: 12-h culture, PMO: 24-h culture, respectively). Samples were immediately pelleted, and treated with RNAprotect Bacteria Reagent (Qiagen, Hilden, Germany). RNA extraction and cDNA conversion were performed

according to the manufacturer's protocol using a NucleoSpin RNA kit (Macherey-Nagel, Duren, Germany) and RNA to cDNA EcoDry Premix (Clontech laboratories Inc.). PCR was carried out using KOD SYBR qPCR Mix (Toyobo, Osaka, Japan) on the CFX96 system (Bio-Rad Laboratories), using the following thermal cycles: 98°C for 2 min, followed by 40 cycles of 98°C for 10 s, 60°C for 10 s, and 68°C for 30 s.

Dot-blot overlay assay. To prepare desialylated PCM, PCM was hydrolyzed with 0.1 M HCl at 80°C for 1 h. Fifty micrograms of PCM or desialylated PCM was blotted onto a nitrocellulose membrane (Bio-Rad Laboratories), followed by blocking with 5% (w/v) BSA-PBS-T. Then, the membrane was treated with biotinylated His-SiaBb2 (40 μg) or lectins (1 μg) in 1% BSA-PBS-T for 3 h at 4°C. After washing the membrane, HRP-labeled streptavidin diluted 1:2,000 in PBS-T was reacted at room temperature for 1 h. Signals were developed with TMB-Blotting Solution (ThermoFisher Scientific, San Jose, CA, USA).

For lectin staining, SSA, MAM, UEA-I, and WGA lectins (0.5 μg, J-OIL MILLS, Inc., Tokyo, Japan) were incubated for 1 h for at 4°C in 1% BSA-PBS-T. After washing the membrane, signals were detected as described above.

Histochemistry-based binding assay. C57BL/6JJcl mice were purchased from CLEA Japan, Inc. (Tokyo, Japan). All mice were housed in SPF conditions at a temperature of 22 ± 2°C under a 12:12 light/dark cycle. Six mice (3 males and 3 females) at 9 weeks of age were anesthetized with Avertin and euthanized by exsanguination. Colonic tissues were excised and fixed, without washing, in methanol in Carnoy's fluid (7). The fixed tissue samples were embedded in paraffin and sectioned at 4-μm thickness. For general morphological observations, Alcian blue (pH 2.5) was used. For His-SiaBb2-staining, deparaffinized sections were incubated with 0.3% H_2O_2 in methanol for 30 min to eliminate intrinsic peroxidase. The sections were rinsed in PBS, blocked with 5% (w/v) BSA-PBS for 1 h, incubated with biotinylated His-SiaBb2 (10 μg) at 4°C for 17 h, reacted with ABC reagent (Vector laboratories, Burlingame, CA, USA) at room temperature for 30 min, and visualized with 3,3'-diaminobenzidine tetrahydrochloride using Histofine Simple Stain MAX-PO kit (Nichirei, Tokyo, Japan). As a control, 5 μg of biotin was used. Pictures were obtained under an Olympus microscope (Tokyo, Japan).

Supplemental references

(1) **Argnani A, Leer RJ, van Luijk N, Pouwels PH**. 1996. A convenient and reproducible method to genetically transform bacteria of the genus *Bifidobacterium*. Microbiology **142**:109–114.

(2) **Hirayama Y**, **Sakanaka M**, **Fukuma H**, **Murayama H**, **Kano Y**, **Fukiya S**, **Yokota A**. 2012. Development of a double-crossover markerless gene deletion system in *Bifidobacterium longum*: functional analysis of the α-galactosidase gene for raffinose assimilation. Appl Environ Microbiol **78**:4984–4994.

(3) **Yasui K**, **Kano Y**, **Tanaka K**, **Watanabe K**, **Shimizu-Kadota M**, **Yoshikawa H**, **Suzuki T**. 2009. Improvement of bacterial transformation efficiency using plasmid artificial modification. Nucleic Acids Res **37**:e3.

(4) **Kiyohara M**, **Tanigawa K**, **Chaiwangsri T**, **Katayama T**, **Ashida H**, **Yamamoto K**. 2011. An exo-alpha-sialidase from bifidobacteria involved in the degradation of sialyloligosaccharides in human milk and intestinal glycoconjugates. Glycobiology **21**:437–447.

(5) **Sillanpää J**, **Martínez B**, **Antikainen J**, **Toba T**, **Kalkkinen N**, **Tankka S**, **Lounatmaa K**, **Keränen J**, **Höök M**, **Westerlund-Wikström B**, **Pouwels PH**, **Korhonen TK**. 2000. Characterization of the collagen-binding S-layer protein CbsA of *Lactobacillus crispatus*. J Bacteriol **182**:6440–6450.

(6) **Turroni F**, **Foroni E**, **Montanini B**, **Viappiani A**, **Strati F**, **Duranti S**, **Ferrarini A**, **Delledonne M**, **van Sinderen D**, **Ventura M**. 2011. Global genome transcription profiling of *Bifidobacterium bifidum* PRL2010 under *in vitro* conditions and identification of reference genes for quantitative real-time PCR. Appl Environ Microbiol **77**:8578–8587.

(7) **Nishiyama K, Sugiyama M, Mukai T**. 2016. Adhesion properties of lactic acid bacteria to intestinal mucin. Microorganisms **4:**E34.
