# Supplementary material for: Bifidobacterium bifidum Extracellular Sialidase Enhances Adhesion to the Mucosal Surface and Supports Carbohydrate Assimilation
Source: mBio. 2017 Oct 3;8(5):e00928-17. doi: 10.1128/mBio.00928-17 (PMC5626965; doi:10.1128/mBio.00928-17)
Supplement: TABLE S1 [file mbo005173503st1.pdf]

SUPPLEMENTAL TABLE

Table S1

Primers used in this study.

Primer	Sequence (5′ to 3′)	Endonuclease*/ Reference
KN149	AAGCTTATTCCCCGCCGAGTCGAAT	*Hind*III
KN150	AAGCTTGAGTCGGCGAAGCCCTT	*Hind*III
KN154	GATAAGCTTTGCTGGATGGGAAGTGG	*Hind*III
KN155	GATAAGCTTTCAGTGAACGGAGCGG	*Hind*III
KN163	ATGGTTCGTTCGACCAAG	
KN165	ATTTTTTTAATCTGTTATTTAAATAG	
KN166	ATGTTTGGATCAGGAGTT	
KN310	GAATGCATATGGCCAGCGATGATGCTGACA	*Nde*I
KN311	AGATTCTCGAGTCAGTGAACGGAGCGGCG	*Xho*I
KN314	GAATGCATATGGCGGACGAAACACCCCAAGAA	*Nde*I
KN315	AGATTCTCGAGTTACCGATTCGCGCGACGGC	*Xho*I
KN318	CATATGAACCTCGGTACCGAATGC	*Nde*I
KN319	CTCGAGCCAATCCATGGAAATGTGCG	*Xho*I
KN325	ACCGGTGGCGGGCGGTC	
KN326	GCCTGTCCATCGGCTC	
uvrD/Rep-F	ATACTCCGAGAATGCGGATG	(1)
uvrD/Rep-R	ACGACATCCCGCTCATATTC	(1)
KNq392	TCGCCGACTCTCAGCTTGGC	
KNq393	GGTGATGTCACGCGGCTTGC	
KN394	GTTAAATTTACATTTTCATTAGTCC	
KN395	AGTTGAGAGTGGACTAAAACC	
KN396	AAGGACGTGACCGTCACGGTGAAT	
KN397	ACACTCACCGTGACCGTCACCA	

*Restriction site is underlined in the sequence.

Reference

(1) **Turroni F**, **Foroni E**, **Montanini B**, **Viappiani A**, **Strati F**, **Duranti S**, **Ferrarini A**, **Delledonne M**, **van Sinderen D**, **Ventura M**. 2011. Global genome transcription profiling of *Bifidobacterium bifidum* PRL2010 under *in vitro* conditions and identification of reference genes for quantitative real-time PCR. Appl Environ Microbiol **77**:8578–858
